# Lactate receptor GPR81 drives breast cancer growth and invasiveness through regulation of ECM properties and Notch ligand DLL4

**DOI:** 10.1186/s12885-023-11631-6

**Published:** 2023-11-22

**Authors:** Kathrine Lundø, Oksana Dmytriyeva, Louise Spøhr, Eliana Goncalves-Alves, Jiayi Yao, Laia P. Blasco, Mette Trauelsen, Muthulakshmi Ponniah, Marc Severin, Albin Sandelin, Marie Kveiborg, Thue W. Schwartz, Stine F. Pedersen

**Affiliations:** 1 Faculty of Health, Novo Nordisk Foundation Center for Basic Metabolic Research, University of Copenhagen, Copenhagen, Denmark; 2 Section for Cell Biology and Physiology, Department of Biology, Faculty of Science, University of Copenhagen, Copenhagen, Denmark; 3 The Bioinformatics Centre, Department of Biology, Faculty of Science, University of Copenhagen, Copenhagen, Denmark; 4 Biotech Research and Innovation Centre, Faculty of Health, University of Copenhagen, Copenhagen, Denmark

**Keywords:** HCAR1, Tumor microenvironment, Spheroid, EPHA7, PCDH7, Notch, Extracellular matrix, Metabolite GPCR

## Abstract

**Background:**

The lactate receptor GPR81 contributes to cancer development through unclear mechanisms. Here, we investigate the roles of GPR81 in three-dimensional (3D) and in vivo growth of breast cancer cells and study the molecular mechanisms involved.

**Methods:**

GPR81 was stably knocked down (KD) in MCF-7 human breast cancer cells which were subjected to RNA-seq analysis, 3D growth, in situ- and immunofluorescence analyses, and cell viability- and motility assays, combined with KD of key GPR81-regulated genes. Key findings were additionally studied in other breast cancer cell lines and in mammary epithelial cells.

**Results:**

GPR81 was upregulated in multiple human cancer types and further upregulated by extracellular lactate and 3D growth in breast cancer spheroids. GPR81 KD increased spheroid necrosis, reduced invasion and in vivo tumor growth, and altered expression of genes related to GO/KEGG terms extracellular matrix, cell adhesion, and Notch signaling. Single cell in situ analysis of MCF-7 cells revealed that several GPR81-regulated genes were upregulated in the same cell clusters. Notch signaling, particularly the Notch ligand Delta-like-4 (DLL4), was strikingly downregulated upon GPR81 KD, and DLL4 KD elicited spheroid necrosis and inhibited invasion in a manner similar to GPR81 KD.

**Conclusions:**

GPR81 supports breast cancer aggressiveness, and in MCF-7 cells, this occurs at least in part via DLL4. Our findings reveal a new GPR81-driven mechanism in breast cancer and substantiate GPR81 as a promising treatment target.

**Supplementary Information:**

The online version contains supplementary material available at 10.1186/s12885-023-11631-6.

## Introduction

A hallmark of solid tumors is increased reliance on glycolysis, caused by tumor hypoxia as well as by the “Warburg effect”, i.e. that many cancers are preferentially glycolytic even in presence of oxygen [1, 2]. Under these conditions, continued glycolysis is dependent on conversion of pyruvate to lactate, allowing the concomitant reconversion of NADH and H^+^ to NAD^+^. Thus, glycolytic cancer cells produce large amounts of lactate, which is extruded by cotransport with protons through monocarboxylate carrier (MCT)-1 and -4 (SLC16A1 and -3) [1, 3]. Tumor cells can also produce lactate via glutaminolysis [4], and stromal cells such as cancer-associated adipocytes are additional sources of lactate [5]. Collectively, this results in lactate concentrations up to 20–40 mM in tumor tissue. High lactate concentration favors tumor growth [6–9] and correlates with poor prognosis in patient tumors [10, 11]. Metabolically, lactate contributes to metabolic symbiosis, in which both cancer- and stromal cells in the tumor can supply oxidative cancer cells with lactate, which is converted to pyruvate and enters the TCA cycle [5]. In addition, lactate functions as a signaling molecule acting through the specific G-protein coupled receptor (GPCR) GPR81, also known as Hydroxycarboxylic Acid Receptor 1 (*HCAR1*) [7, 12–14]. Under normal physiological conditions, GPR81 is expressed in certain immune cells [8], but otherwise mainly in adipocytes. In adipocytes, GPR81 functions as an autocrine sensor of lactate produced by aerobic glycolysis, acting at least in part via a G_i_-dependent decrease in cAMP to inhibit lipolysis [15, 16]. It was however, recently demonstrated that GPR81 is surprisingly highly expressed in cancer cells of many different types of solid tumors [8] and that GPR81 knockdown (KD) reduced growth of breast [17] and pancreatic [18] tumor xenografts in immunosuppressed mice. Furthermore, GPR81 depletion in various cancer cells reduced expression of MCT1 and -4 and limited cancer cell growth, invasiveness, chemotherapy resistance, and Programmed death-ligand 1 (PD-L1) expression [17–23]. Recent work has proposed that GPR81 regulates immune cell infiltration in breast cancers [9, 24]. However, the mechanisms through which GPR81 exerts these effects remain incompletely understood.

The aim of this study was to identify mechanisms through which GPR81 regulates cancer cell 3D growth and invasiveness. GPR81 was upregulated by lactate and in 3D culture, and GPR81 KD increased spheroid necrosis, and inhibited migration, invasion and in vivo tumor growth. Importantly, in MCF-7 breast cancer cells as a model of Luminal A subtype breast cancers, GPR81-regulated genes were significantly associated with multiple GO terms relating to extracellular matrix (ECM) and cell adhesion, including PCDH7, EPHA7, and the Notch ligand DLL4. We conclude that GPR81 upregulation in the tumor microenvironment supports breast cancer aggressiveness at least in part via DLL4 upregulation and remodeling of ECM composition and cell–cell and cell–matrix interactions. Our findings reveal a new GPR81-driven mechanism in Luminal A breast cancer and substantiate GPR81 as a promising treatment target.

## Methods

### Cell lines and cell culture

MCF-7 (ATCC, #HTB-22) and MDA-MB-231 (ATCC, #HTB-26) were grown in DMEM supplemented with 1% non-essential amino acids (NEAA) (#M7145, Sigma-Aldrich), 1% Penicillin/Streptomycin (pen/strep) (#069, SSC, University of Copenhagen) 2 mM glutamine and 10% FBS. T47D cells (ATCC, #HTB-133) were grown in RPMI 1640 (#075, SSC, University of Copenhagen) supplemented with 10% FBS and 0.5% Insulin-Transferrin-Selenium (#41400–045, Gibco) and 1% pen/strep. SKBr-3 cells (ATCC-HTB-30) were grown in McCoy’s 5a Modified Medium (#M9309, Sigma) supplemented with 10% FBS and 1% pen/strep. MCF10A cells (ATCC, #CRL10-317) were grown in a 1:1 mix of DMEM (#41966, Gibco) and Ham’s F12 nutrient mixture medium (#N6658, Sigma) supplemented with 1% pen/strep, 5% FBS, 20 ng/ml recombinant human epidermal growth factor (#E9644, Sigma), 0.25 ng/mL hydrocortisone (#H0888, Sigma), and 10 µg/ml bovine insulin-transferrin-selenium (#41400–045, Gibco).

### Lactate / glucose treatment

Extracellular lactate concentrations of 10–30 mM have been reported in patient tumors, compared to 1.5–3 mM in normal tissues [11, 25]. Although such measurements are subject to technical challenges [26], we therefore chose 20 mM lactate for mimicking tumor concentrations in our experiments. Cells were seeded and grown in standard growth medium as above overnight, and the next morning, the medium was changed to either glucose- and pyruvate-free DMEM (#A14430-01, Gibco) supplemented with 20 mM Na^+^-lactate (#L7022-10G, Sigma), 2% FBS, and 1% NEAA (*lactate medium*) or medium of the same composition but with 5 mM *D*-glucose (#G7021-1 KG, Sigma) instead of Na^+^-lactate (*glucose medium*).

### RNA extraction and qPCR analysis

RNA was prepared using the RNeasy Micro Kit (#74004, Qiagen) according to the manufacturer’s instructions. cDNA was prepared using Superscript III Reverse Transcriptase (#18080–085, Invitrogen). RT-PCR was performed using PrecisionPLUS MasterMix SYBRgreen (#PPLUS-1ML, Primer Design). ATCB, GAPDH and TBP were used as reference genes. Primer sequences are listed in Suppl. Table 8. Relative gene expression was calculated using the ΔΔCt-method.

### siRNA- and shRNA-mediated knockdown experiments

siRNA information is found in Suppl. Table 9. siRNA transfections were performed according to manufacturer’s instructions. GPR81 RNA oligonucleotide (siGPR81, 5’-GAAGAGAUGCCAAUUUCGA-3 (fw), 5- ‘UCGAAAUUGGCAUCUCUUC-3’ (rv) and siControl, MISSION® siRNA Universal Negative Control #1 were used (#SIC001, Sigma Aldrich). siRNA to a final concentration of 0.5 ng/ml was mixed with Lipofectamine 3000 (#L3000015, ThermoFisher) according to manufacturer’s instructions. Oligomer-Lipofectamine complexes were then gently added to the cells. Oligomer-Lipofectamine complexes were gently added to the cells and left to incubate for 48 h before assays at 37°C, 5% CO_2_.

shRNA transfection was performed using HCAR1 Mission shRNA Bacterial Glycerol Stock (SHCLNG_NM_032554, Sigma Aldrich (NM_032554.2-1217s1c1 for construct #1 and NM_032554.2-2200s1c1 for construct #2); Suppl. Table 9). Plasmids were generated according to manufacturer’s protocol and extracted using NucleoBond Xtra Midiprep (#740410.50, Macherey–Nagel). 5 µg shRNA was mixed with Lipofectamine 3000 and P3000TM Enhancer Reagent (#L3000015, ThermoFisher) according to manufacturer’s instructions. Oligomer-Lipofectamine complexes were gently added to the cells by drops. 24 h after transfection, the cells were selected using 1 µg/mL Puromycin (#A11138-03, Gibco) and a parallel dish of non-transfected cells which were 100% killed by adding puromycin as a control. Medium was changed every 2–3 days. Transfected cells were cultured in DMEM (5% FBS, 2 mM Glutamine, 1% NEEA, 1% pen/strep, 2.5 µg/mL puromycin).

### Agarose fixation and paraffin embedding of spheroids

Spheroids were isolated and fixed in 4% PFA for 24 h prior to embedding. An agarose gel was prepared using Bacto™ Agar (#214050, BD) diluted in MiliQ water and heated until fully melted. The agarose was put on Superfrost Ultra Plus Object Glasses (#J380AMNZ, Thermo Fisher) in drops and 15–20 spheroids were then injected into the center of the drop and left to solidify. The spheres were put into cassettes and submerged into 96% ethanol for 30 min, 99% ethanol for 2 × 30 min. Subsequently, they were submerged in xylene for 2 × 30 min before being embedded in paraffin.

### In situ hybridization and immunohistochemistry

Paraffin-embedded spheroids were cut into 5 µm thin cross-sections using a Microtome HM200 and mounted onto Superfrost slides. The sections were baked at 60°C for 60 min, deparaffinized and rehydrated. Slides were air-dried at room temperature before continuing with a hydrogen peroxide pre-treatment for 10 min and target retrieval by boiling in target retrieval reagent for 5 min. Protease plus (#322330, Advanced Cell Diagnostics) was added for tissue permeabilization and slides were incubated for 15 min at 40°C. Then tissue was hybridized with chosen probes (Suppl. Table 11) using Multiplex Fluorescent detection reagents v2 (#323110, Advanced Cell Diagnostics) according to the manufacturers protocol. Probe signals were developed by incubation with Opal reagents (Akoya Bioscience). The slides were incubated with blocking buffer (5% donkey serum in PBS) at room temperature for 45 min. Primary antibodies were added and slides were incubated overnight at 4°C. Slides were washed in 1 × PBS five times before adding secondary antibodies (1:800 in 5% donkey serum). Slides were incubated for 1 h at room temperature before washing in PBSand mounted with ProLong™ Gold Antifade Mountant with DAPI (#P36935, Invitrogen). Images were acquired using Zen Pro 3.0 Software connected to a Zeiss Axiocam 702 monochrome microscope camera using a 20X/0.8 NA objective. Expression of chosen markers was evaluated using the HALO image analysis platform which detects intensity of expression in each cell. Cells were divided into 5 bins (0 to 4), where bin 0 contain cells with no detectable expression, bin 1 contains cells with 1–3 dots, bin 2, cells with 4–9 dots, bin 3, cells with 10–15 dots, and bin 4, cells with more than 15 dots.

### Cell viability assay

The effect of GPR81 KD on cell viability was determined using CellTiter-Glo Luminescent Cell Viability Assay (#G7570, Promega) according to manufacturer’s instructions. Cells were grown under normal conditions overnight and the medium was changed to either 20 mM lactate medium (0 mM glucose, 2% FBS, 1% NEEA) or 5 mM glucose medium (0 mM lactate, 2% FBS, 1% NEEA) and incubated for 72 h before assessing luminescence (RLU).

### Migration and invasion assay

Cells were incubated with lactate medium 24 h prior to experiments. Boyden chamber culture inserts (#353097, Corning) with 8 µm pores were covered with 3% matrigel (for invasion) (#354234, Corning) by adding 100 µL matrigel per insert and allowing it to polymerize at 37°C for 1 h. Cells were seeded at 50,000 cells in 0.1% FBS lactate medium (DMEM with 20 mM lactate and 1% pen/strep, 1% NEAA, 0 glucose, 0 glutamine, 0 phenol red). For invasion, cells were stimulated with 50 nM 17-β-estradiol (#E2257, Sigma) upon seeding. The lower compartment contained lactate medium as above but containing 10% FBS as a chemoattractant. Chambers were incubated for 24 h at 37°C, washed in cold PBS, and remaining cells were gently removed from the apical side with a cotton swab. Membranes were fixed in 4% PFA for 20 min and washed 3 times in PBS, followed by permeabilization with 0.5% Triton X-100 for 20 min. Cells were stained and mounted using ProLong™ Gold Antifade Mountant (#P10144, Thermo Fischer), imaged using a 10X/0.3 NA objective and an Axio Observer microscope (Zeiss) and quantified in ImageJ (version 1.8).

### Western blotting

Cells were lysed in 95°C SDS lysis buffer (0.1 M Tris–HCl, 0.1 M pH 7.5, 1% SDS, 1 mM Na_3_VO_4_ and Complete™ protease inhibitor (#11836153001, Roche). Lysates were homogenized by sonication (PowerMED), centrifuged (Micromax RF, Thermo) for 5 min at 20,000 × g at 4°C, and protein concentrations determined using the DC Protein assay kit (#500–0113, #500–0114, #500–0115, BioRad). Samples were normalised with ddH_2_O and mixed with NuPAGE LDS 4 × sample buffer (#NP0007, Invitrogen) and dithiothreitol (DTT). Equal amounts of protein were separated by SDS-PAGE using Criterion 10% Tris gels (Bio-Rad) and Tris/Glycine/SDS running buffer (#161–0732, BioRad), and BenchMark protein ladder (#10747–012, Invitrogen). Proteins were transferred using the Trans-Blot Turbo transfer system (BioRad) to Trans-Blot Turbo 0.2 μm nitrocellulose membranes (#170–4159, BioRad). Membranes were stained with Ponceau S (#P7170-1L, Sigma-Aldrich), blocked for 1 h at 37 °C in 5% nonfat dry milk in TBST (0.01 M Tris/HCl, 0.15 M NaCl, 0.1% Tween 20, pH 7.4). Membranes were incubated with primary antibodies (Suppl. Table 10) diluted in 5% nonfat dry milk in TBST overnight at 4°C, and with horseradish peroxidase (HRP) conjugated secondary antibodies in 5% nonfat dry milk in TBST for 1 h at room temperature. Bands were developed by enhanced chemiluminescent (ECL) substrate (#32106, Pierce) or SignalFire (#6883, Cell Signaling) and visualized with Fusion Fx (Vilber Lourmat). Densitometric analyses were carried out using ImageJ. No grouping or splicing of Western blots was performed.

### Adhesion assay

Cells were seeded in 6-well plates with 20 mM lactate medium and incubated 24 h at 37 °C, 5% CO_2_. A 96-well plate was coated with Matrigel (#354234, Corning) for 1 h at 37 °C and with 10 mg/mL BSA for 1 h at room temperature. Cells were plated at 60,000 cells/well and incubated 1 h at 37°C to ensure attachment to the matrigel. Cells were washed in PBS, fixed in ice cold methanol for 30 min at room temperature, and washed 3 times in PBS before staining with 0.1% crystal violet for 30 min at room temperature. Cells were washed 3 times with ddH_2_O and completely dried. Images were acquired using a 5X/0.16 NA objective and an Axio Observer microscope (Zeiss). Crystal violet was solubilized in 10% acetic acid, and absorbance was measured at 570 nm using a CLARIOstar platereader (BMG Labtech). Images were quantified using ImageJ version 1.8 to determine %area fraction.

### Wound healing assay

Cells were plated in 2-well culture inserts (#80209, Ibidi Gmbh) in 24-well plates at 1.5 × 10^5^ cells/well, treated with 5 µM aphidicolin (#A4487, Sigma) to inhibit cell proliferation, and incubated overnight. Culture medium was changed to 20 mM lactate medium 24 h prior to the assay. After overnight incubation the inserts were removed, cells were washed once in PBS and fresh lactate medium was added. Images were acquired at timepoints 0, 2, 4, 6, 8, 24 and 48 h with a 5X/0.16 NA objective and an Axio Observer microscope (Zeiss). The gap areas for each image were calculated using ImageJ (Version 1.8) and used to determine the % closure of the gaps at each timepoint.

### Immunocytochemistry

Cells were cultured in 6-well glass bottom culture dishes for 48 h, 37°C, 5% CO_2_. Growth medium was replaced by 20 mM lactate medium and cells further incubated for 24 h. Cells were fixed in 2% PFA (#47608, Sigma) for 15 min at room temperature followed by 30 min on ice, and washed twice in TBST. Cells were permeabilized using 0.5% Triton-X-100 (#9002–93-1, Sigma) for 5 min and blocked in 5% BSA (#A7906, Sigma) in TBST (TBS + Tween20 (#P1379, Sigma) for 30 min. Cells were incubated overnight at 4°C with primary antibodies (Suppl. Table 10) diluted in TBST + 1% BSA, then washed 3 times in TBST + 1% BSA before incubation with secondary antibodies diluted in TBST + 1% BSA for 1 h. Cells were washed in TBST + 1% BSA, incubated with DAPI diluted in TBST + 1% BSA for 5 min, and washed once in TBST + 1% BSA. Coverslips were mounted using N-propyl gallate antifade. Cells were imaged using the 60X objective of an Olympus IX83 microscope system and data processed using ImageJ.

### 3D spheroid culture

Cells were seeded at 1000 cells/well in round-bottomed, ultra-low attachment plates (#7007, Corning) and grown for 7–14 days at 37°C. Media for MCF10A and MDA-MB-231 cells were supplemented with 1.5% Geltrex LDEV-Free Reduced Growth Factor Basement Membrane Matrix (#A1413202, Thermo-Fisher). Plates were centrifuged for 15 min, 750 RCF after seeding.

Images were acquired using a 10X/0.3 NA objective and an Axio Observer microscope (Zeiss), on days 2, 4, 7, 9, 11 and 14. To quantify spheroid growth, spheroids were measured using the free-hand drawing function in ImageJ, with each data point representing the mean of 5–10 spheroids per condition.

### In vivo tumor model

Eight week old NOD.Cg-Prkdcg Il2rg^tm1Wjl^ / SzJ female mice (Charles River Wilmigton, MA, USA) were randomly assigned to inoculation with 0.25 × 10^6^ MDA-MB-231 pLKO.1 or shGPR81 cells in 4th mammary fat-pad. 10 mice were inoculated per group. Animals were anesthetized by inhalation of isoflurane and cells were inoculated subcutaneously. Tumor size was evaluated three times/week using a digital caliper (Silvan, DK), and tumor volume (V) was calculated as: V = (LxW2)/2, where L is tumor length and W is tumor width. Tumors were excised when they reached a volume of 550–600 mm^3^ or for ethical reasons (ulcers or loss of weight). Animals were sacrificed by cervical dislocation without the use of anesthesia. Animal experiments were carried out in accordance with ethical regulations from the Danish government and approved by the Danish Veterinary and Food administration (license #2019–15-0201–01642). The study is reported according to ARRIVE guidelines.

### RNA-seq analysis and GO enrichment analysis

RNA was prepared as above, with 4 replicates per group. RNA-seq was performed by BGI, Hong Kong. RNA-seq reads were pseudo-aligned to Gencode transcriptome release 34 and quantified using Salmon v1.1.0 [27]. Pre-filtering for low counts was performed using the filterByExpr function in the edgeR package with parameter min.total.count = 10 [28, 29]. TMM normalization [30] and room transformation [31] were applied with a design matrix that used the intersection of cell type and medium as coefficient, namely shGPR81_lactate, shGPR81_glucose, pLKO.1_lactate, and pLKO.1_glucose. Differential expression (DE) analyses were performed using the following contrasts: shGPR81_lactate vs pLKO.1_lactate, and shGPR81_glucose vs pLKO.1_glucose using makeContrasts function in the Limma R package [32]. DE, given the above settings, was defined as Benjamini–Hochberg *FDR* < 0.05 and an absolute log_2 _fold change > 0.5.

Gene ontology (GO) enrichment analyses were performed on DE genes using gProfiler2 [33]. The background gene set was assigned to all genes expressed under the pre-filtering threshold as defined above. Significance threshold was set to Benjamini–Hochberg *FDR* < 0.05.

### Statistical analysis

Unless otherwise indicated, data are shown as means with standard error of the mean (SEM) error bars or as representative images, and statistical analysis was performed in Graphpad Prism (Version 8). Two-way ANOVA and Sidak’s multiple comparisons post-test was used for > two groups, and paired Student’s *t*-test or one-way ANOVA when two groups were compared. In vivo tumor growth was analysed using a Mann–Whitney test. *P* < 0.05 was considered statistically significant.

## Results

### GPR81 is differentially upregulated in tumor tissues, and high GPR81 expression correlates with poor survival in Luminal A breast cancer patients

**Fig. 1:**
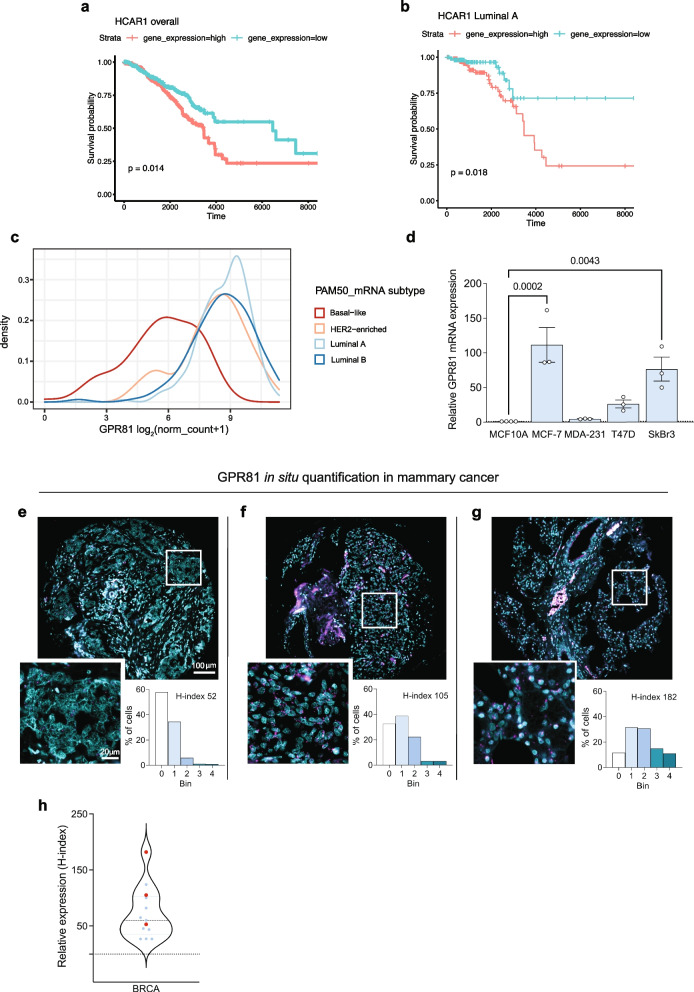
GPR81 mRNA expression is upregulated in tumor tissues and cell lines. **a**-**b** Kaplan–Meier analysis of patient overall survival as a function of GPR81 (HCAR1) expression. **a** All 1194 breast cancer patient data sets in TCGA. **b** Luminal A subtype (222 patients) after stratification by PAM50 subtype. The corresponding data for Luminal B, Basal and HER2 enriched subtypes are found in Suppl. Figure 1. **c** Density plot shows GPR81 (HCAR1) expression in breast cancer patients of PAM50 subtypes from TCGA data. Luminal A subtype breast cancer tissue has the highest, and triple-negative breast cancer (TNBC) tissue has the lowest GPR81 expression. **d** qPCR analysis of relative GPR81 mRNA expression in human normal mammary epithelial cells (MCF10) and human mammary cancer cell lines (*n* = 3 independent replicates per cell line, indicated by dots). **e**–**g** In situ RNAscope analysis of GPR81 expression, quantified as the H-index, a measure of GPR81 expression and heterogeneity. The figure shows examples and corresponding bin quantifications for three breast cancer cores with low, medium and high overall GPR81 H-index. The H-index was calculated by totalling % cells in each bin, according to a weighted equation where bin 0 corresponds to 0, bin 1 to 1, etc., after grouping of cells into 5 bins (groups) based on the number of dots per cell. Each sample was evaluated for the % cells in each bin (see Materials and Methods). The Y-axis shows the % of cells with a given bin distribution. **h** All RNAscope analyses of GPR81 expression in human patient breast cancer tissue. Each dot corresponds to a biopsy from single patient, with the red dots corresponding to the analyses shown in panel **e**–**g**. In Suppl. Figure 1 h, these data are compared with similar analyses from other tumor types to illustrate the heterogeneity of GPR81 expression

Analysis of human tumor and control tissue data from the TCGA and GTeX databases [34, 35] showed that the mRNA level of GPR81 (i.e. *HCAR1*) is increased or tends to be increased in many cancers, including pancreatic, ovarian, bladder, colon, and lung cancer, compared to the corresponding control tissue (Suppl. Figure 1a). Both normal mammary tissue and breast cancer tissue exhibited high expression compared to other tissues, presumably reflecting the contribution from adipose cells to GPR81 expression in this tissue. Importantly however, Kaplan-Meyer analysis of TCGA data showed that breast cancer patient survival negatively correlates with tumor GPR81 mRNA expression (Fig. 1a). When data were stratified by PAM50, the correlation was only significant for the Luminal A patient group (Fig. 1b, Suppl. Figure 1b-e). Consistent with this, further analysis of TCGA data revealed that Luminal A subtype cancer tissue has the highest, and triple negative breast cancer (TNBC) tissue the lowest, GPR81 expression (Fig. 1c). A similar pattern was observed by qPCR analysis of breast cancer cells in culture, with MCF-7 cells (luminal A) having the highest, and MDA-MB-231 cells (TNBC) and non-transformed MCF10A cells having the lowest, GPR81 expression (Fig. 1d). Similarly, high GPR81 expression was detected in most human pancreatic, bladder, colon, lung cancer cell lines studied as well as in several mouse cancer cell lines (Suppl. Figure 1f-g).

Detailed understanding of GPR81 expression, and in particular its spatial organization in tumors, has been limited by the lack of specific antibodies [36].^1^ To evaluate GPR81 distribution within tumors, we therefore performed in situ hybridization in tissue microarrays (TMAs) of patient tumor biopsies, having established that the in situ probes were highly specific for GPR81 (Suppl. Figure 2). The in situ hybridization data were quantified as the *H-index*: for each tumor, cells were grouped into 5 *bins* (groups) with bin 0 having the lowest, and bin 4 the highest, number of GPR81 dots (Fig. 1e-g, bar charts). The H-index was calculated as the weighted sum of these bins. Figure 1e-g shows representative in situ staining patterns and corresponding quantifications for three breast cancer tumors with overall H-indices of 52, 105, and 182. Figure 1h summarizes shows H-index data from the in situ analysis of breast invasive carcinomas, with the H-indices from the three examples marked with red dots. Suppl. Figure 1 h compares the breast cancer H-indices with similar analyses from pancreatic adenocarcinoma, ovarian cancer, bladder cancer, colon and lung cancer patient tumor tissue. The large span of H-index values indicates that GPR81 expression in tumor tissue is highly variable also between patients within each cancer type.

Collectively, these data show that GPR81 expression is upregulated with high incidence in tumors from many cancer types, varies with breast cancer subtype, is heterogeneously expressed within tumor tissues, and correlates with poor survival in breast cancers, especially of the luminal A subtype.

### GPR81 is upregulated by high extracellular lactate and 3D spheroid growth of breast cancer cells

**Fig. 2:**
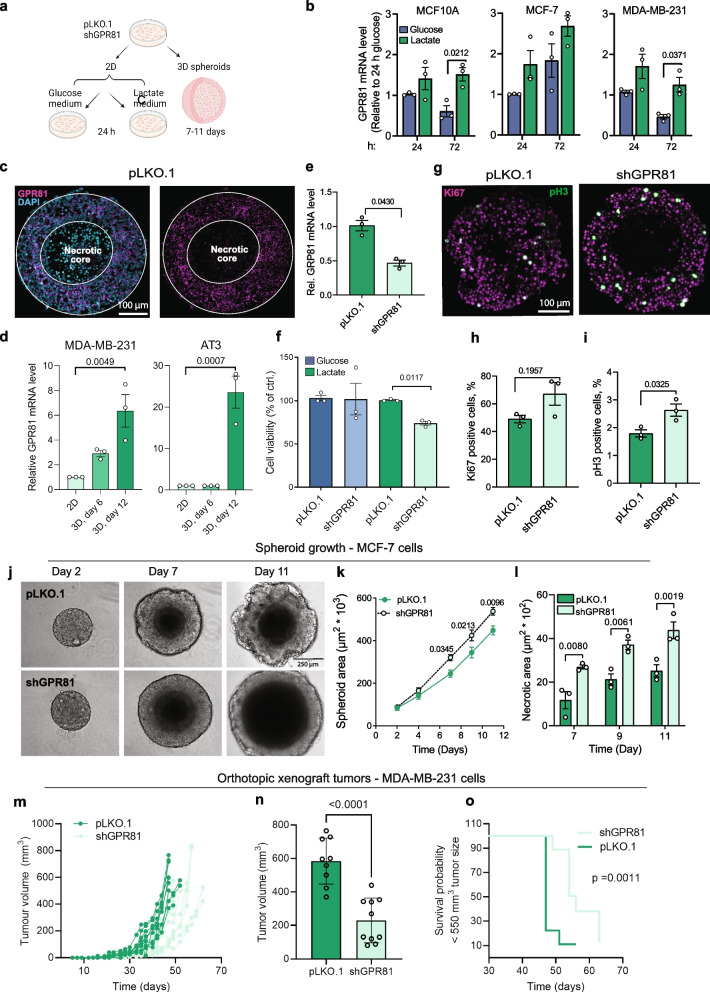
GPR81 is upregulated by extracellular lactate and by 3D spheroid growth and GPR81 KD inhibits 2D growth and increases 3D necrosis of breast cancer cells. **a** Experiment overview. **b** qPCR analysis of relative GPR81 mRNA levels in 2D cultures of breast epithelial (MCF10A) and breast cancer cells (MDA-MB-231, MCF-7) cultured for 24 and 72 h with glucose (5 mM glucose/0 mM lactate) or lactate (20 mM lactate/0 mM glucose) medium. *n* = 3 independent replicates per cell line, two-way-ANOVA with Sidak Post Hoc test. **c** Representative in situ analyses of GPR81 expression in MCF-7 control (pLKO.1) and GPR81 knockdown (shGPR81) spheroids. *n* = 3. **d** qPCR analysis of GPR81 mRNA levels in 2D vs day 6 and 12 3D cultures of MDA-MB-231 and AT3 cells (*n* = 3, paired, two-sided Student’s *t*-test). **e** Relative GPR81 expression in MCF-7 control (pLKO.1) and GPR81 KD (shGPR81) cells, *n* = 3, paired, two-sided Students *t*-test. **f** Viability of MCF-7 GPR81 KD cells. Cells were incubated in glucose or lactate medium for 72 h before assessing cell viability. Data is presented as % cell viability compared to control (pLKO.1) (*n* = 3, paired, two-sided Students *t*-test). **g**-**i** Representative images (**g**) and corresponding quantification (**h**-**i**) of MCF-7 pLKO.1 and GPR81 KD (shGPR81) spheroids stained for Ki67 and phospho-Histone H3 (pH3). Graphs show % live cell positive for Ki67 (H) or pH3 (I). (*n* = 3, paired, two-sided Student’s *t*-test). **j** Spheroid growth of MCF-7 cells. Representative brightfield images taken on day 2, 7 and 11. Scalebar: 250 µm. **k** Spheroid area. **l** Necrotic core area (µm^2^ * 10^3^) relative to spheroid size on day 7, 9 and 11. (*n* = 5). Two-way ANOVA with Tukey post-test. **m**–**o** Orthotopic xenograft model using MDA-MB-231 GPR81 KD cells. Eight-week-old female NOD.Cg-Prkdcg Il2rg^tm1Wjl^/SzJ mice were inoculated with 0.25 × 10^6^ pLKO.1 or shGPR81 cells (*n* = 10 per condition). Tumor volume was evaluated three times per week after cell inoculation. **m** Individual tumor growth curves for pLKO.1 and shGPR81 xenografts. **n** Tumor volume (mm^3^) on day 47. **o** Kaplan–Meier overall survival of mice bearing pLKO.1 or shGPR81 xenografts. Mice were sacrificed when they reached a tumor volume of 550–600 mm^3^ or had tumor ulcers. Log-rank statistics was used to compare statistical significances between groups (*p* = *0.0014*)

In non-cancer human breast epithelial cells (MCF10A) and in triple-negative (MDA-MB-231) breast cancer cells, GPR81 mRNA expression was higher in lactate-rich medium (20 mM lactate, 0 mM glucose) than in normal glucose (5 mM) medium, after 72 h (Fig. 2a-b), confirming earlier findings in pancreatic [18] and lung [37] cancer cells. A similar trend was seen for Luminal A (MCF-7) breast cancer cells (Fig. 2b).

In growing tumors, lactate concentrations increase due to the combination of high glycolytic metabolism and poor perfusion. To mimic tumor microenvironment conditions, breast cancer cells were next cultured as 3D spheroids. In situ hybridization revealed that GPR81 was predominantly expressed in non-necrotic spheroid cells close to the necrotic core, with lower expression in cells in the spheroid periphery (Fig. 2c). Notably, this distribution is highly reminiscent of the distribution of lactate in spheroids [38]. We therefore asked whether 3D spheroid growth was associated with increased GPR81 expression. Spheroids were harvested for qPCR analysis on day 7 and GPR81 expression compared with that in parallel 2D cultures. Except for MCF-7 cells which have very high GPR81 expression already in 2D culture, all spheroids exhibited higher GPR81 expression than corresponding 2D cultures (Suppl. Figure 3a). Marked, time-dependent GPR81 upregulation was observed in 3D culture, most evident in cell types which express the receptor at relatively low levels in 2D, i.e. MDA-MB-231 and the murine AT-3 breast cancer cell line (Fig. 2d).

These results show that GPR81 expression is increased by high extracellular lactate in 2D culture, and by 3D growth as another means of increasing microenvironmental lactate.

### GPR81 knockdown in breast cancer cells inhibits 2D growth, increases 3D necrosis, and delays in vivo tumor growth

We next analysed the importance of GPR81 for cancer cell growth under 2- and 3D conditions. GPR81 KD, either transiently (Suppl. Figure 3b-c) or by stable shRNA transfection (Fig. 2e-f), had no effect on MCF-7 cell viability in glucose medium, yet reduced viability with lactate as the nutrient source in 2D culture. Hyperphosphorylated retinoblastoma protein (p-pRb), indicative of proliferative capacity, was reduced in lactate- compared to glucose medium but was unaffected by GPR81 KD (Suppl. Figure 3d-e), suggesting that the role of GPR81 was mainly related to survival rather than to proliferation.

Proliferation was also not strongly affected by GPR81 KD in the 3D setting, where protein levels of Ki67 (proliferation marker) and phospho-Histone 3 (pH3, mitosis marker) were modestly increased after GPR81 KD in MCF-7 spheroids (Fig. 2g-i) and unaffected in MDA-MB-231 spheroids (Suppl. Figure 3 h-j). Importantly, after GPR81 KD, Ki67 and pH3 staining was exclusively found at the periphery of MCF-7 spheroids, which appeared more circular than that of pLKO.1 controls, with a markedly expanded necrotic core (Fig. 2g). Further analysis of MCF-7 spheroid morphology and growth over time confirmed this and demonstrated that GPR81 KD spheroids grew slightly larger than corresponding controls, yet with a characteristically tighter, more circular appearance and an expanded necrotic core (Fig. 2j-l). This suggests that cell–cell adhesion was increased upon loss of GPR81, limiting access to nutrients and oxygen and preventing venting of waste products. This will eventually lead to necrotic cell death, a phenomenon well described in such regions of tumors and in spheroid models [39].

Collectively, these findings are fully consistent with the previously reported finding that stable shRNA-mediated KD of GPR81 in MCF-7 cells substantially reduces tumor xenograft growth in immunosuppressed mice [17].

GPR81 has previously been shown to regulate the expression of lactate-H^+^ cotransporters of the SLC16 family [18]. Consistent with this, KD of GPR81 in MCF7 cells tended to reduce the mRNA expression of the lactate-H^+^ cotransporter MCT4 (SLC16A3) but not that of MCT1 (SLC16A1) (Suppl. Figure 3i-j). MCT4 is particularly well suited to will facilitate lactate extrusion under conditions of high anaerobic glycolysis, and its upregulation is associated with poor prognosis in many cancers [40].

Because MDA-MB-231 cells are more mesenchymal and MCF-7 cells more epithelial, MDA-MB-231 spheroids have a much looser organization than do MCF-7 spheroids, with little necrotic core development (see also [41]). In MDA-MB-231 spheroids, transient GPR81 KD tended to reduce spheroid growth while GPR81 overexpression had no additional effect (Suppl. Figure 3 k-l). To further validate these findings, we next assessed the effect of GPR81 KD on the growth rate of orthotopic MDA-MB-231 tumors in NSG mice. On day 47 after inoculation, tumor volume was reduced by over 50%, with a mean tumor volume of ~ 230 mm^3^ in the GPR81 KD group, compared to ~ 550 mm^3^ in the control group (Fig. 2m-n). Similarly, event-free survival, with event defined as reaching a tumor size of 550 mm^3^, was significantly increased (*p* = 0.0011) by GPR81 KD (Fig. 2o). Mice were terminated when tumor size reached 550–600 mm^3^, at which time there were no detectable macroscopic differences between control- and GPR81 KD tumors (Suppl. Figure 4a-b).

These findings show that during 3D- or in vivo growth, i.e. conditions of high extracellular lactate, breast cancer cells are dependent on GPR81 for growth.

### GPR81 stimulates breast cancer cell adhesion, migration, invasion, and Akt activity

**Fig. 3:**
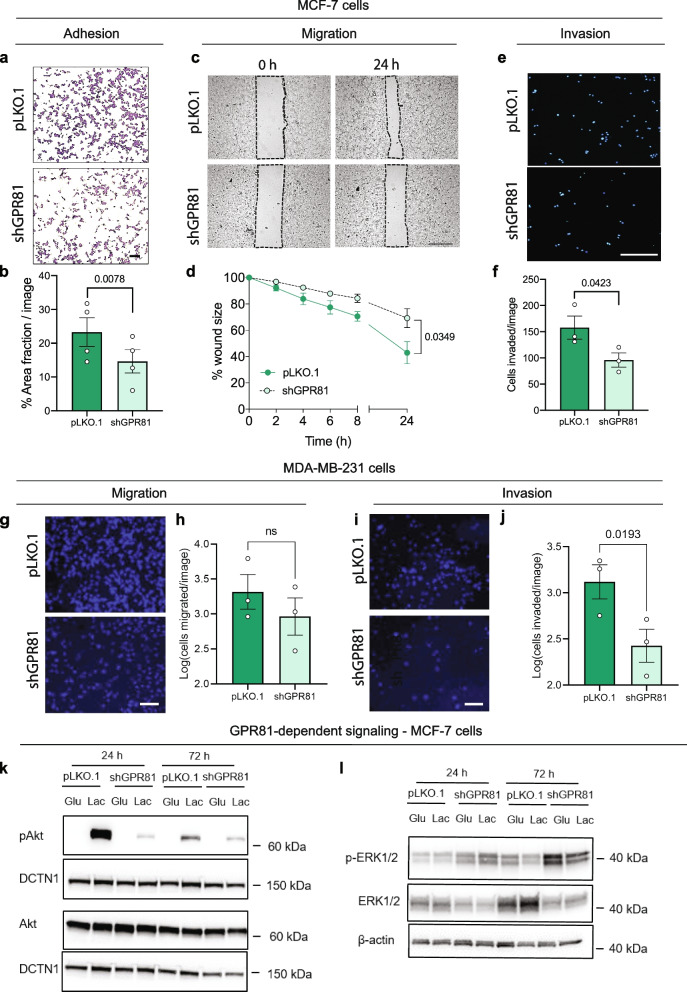
GPR81 stimulates breast cancer cell adhesion, migration, invasion, and Akt activity. **a**-**b** Adhesion of MCF-7 cells to matrigel is reduced by GPR81 KD. Cells were incubated for 24 h in lactate medium prior to being seeded on matrigel. After 1 h, nonadherent cells were washed of, and cells were fixed and stained. **a** Representative images (*n* = 4). **b** %Area-fraction/image, i.e. the relative area covered by cells. **c**-**d** Migration of MCF-7 cells is reduced by GPR81 KD. **c** Cells were seeded in lactate medium in Ibidi insert plates and treated with aphidicolin to block proliferation. Inserts were removed after 24 h, and images were acquired at the time points shown (*n* = 3). Scalebar: 500 µm. **d** Average wound size as % of size at t=0, normalized to pLKO.1 cells. **e**–**f** Invasion of MCF-7 cells through matrigel is reduced by GPR81 KD. Cells incubated in lactate medium for 24 h were seeded in the upper chamber of matrigel-coated Boyden chambers. The lower chamber contained the same medium plus 10% FBS. After 24 h, membranes were processed for imaging. **e** Representative images (*n* = 3). Scalebar: 500 µm. **f** Cells invaded/image. **g**-**j** Boyden chamber analysis of migration (**g**-**h**) and invasion (**i-j**) of MDA-MB-231 pLKO.1 and shGPR81 cells. **g,i** Representative images of migration (**g**) and invasion (**i**), **h,j** Migrated/invaded cells per image. Scale bars: 100 µm. **k**-**l** Representative Western Blots of active, Ser473-phosphorylated Akt (p-Akt) and total Akt (**k**), and active, Thr202/Tyr204-phosphorylated ERK1/2 and total ERK1/2 (**l**) in pLKO.1 and shGPR81 MCF-7 cells, following incubation in glucose- or lactate medium for 24 or 72 h. DCTN1 and β-actin serve as a loading controls. Statistics (**b, d, f, h, j**): Paired, two-sided Students *t*-test

Reduced cell–cell adhesion and increased cell–matrix adhesion are traits characteristic of the epithelial-to-mesenchymal (EMT) transition associated with increased cancer cell motility and metastasis [42]. The striking increase in circularity and tightness of MCF-7 spheroids induced by GPR81 KD (Fig. 2j) suggested to us that GPR81 activity could inhibit cell–cell adhesion. We therefore asked whether other EMT traits were also attenuated by loss of the receptor. Consistent with this notion, MCF-7 cell adhesion on matrigel (Fig. 3a-b), migration (Fig. 3c-d), and invasion through matrigel (Fig. 3e-f) were all inhibited by GPR81 KD. Similar effects were observed in MDA-MB-231 cells, in which GPR81 KD tended to inhibit migration (Fig. 3g-h) and inhibited invasion (Fig. 3i-j), fully in line with findings in a recent report [43].

Signaling via Ser/Thr kinases Akt and extracellular signal regulated kinase-1/2 (ERK1/2) plays central roles in cancer cell adhesion and motility [44, 45]. To assess their possible regulation by GPR81, the activity of these kinases was evaluated by immunoblotting in control cells and after GPR81 KD. 24 and 72 h of lactate exposure potently increased activating phosphorylation of Akt1/2 without affecting total Akt protein level. Importantly, lactate-induced Akt phosphorylation was abolished by GPR81 KD (Fig. 3k). In marked contrast, activating phosphorylation of ERK1/2 was increased by GPR81 KD, while total ERK1/2 expression was decreased (Fig. 3l).

Collectively, these results show that GPR81 favors breast cancer cell adhesion, migration and invasion, Akt activation and ERK1/2 protein expression, while limiting ERK activation.

### GPR81 KD in MCF-7 cells alters expression of genes involved in cell adhesion, ECM regulation and Notch signaling

**Fig. 4:**
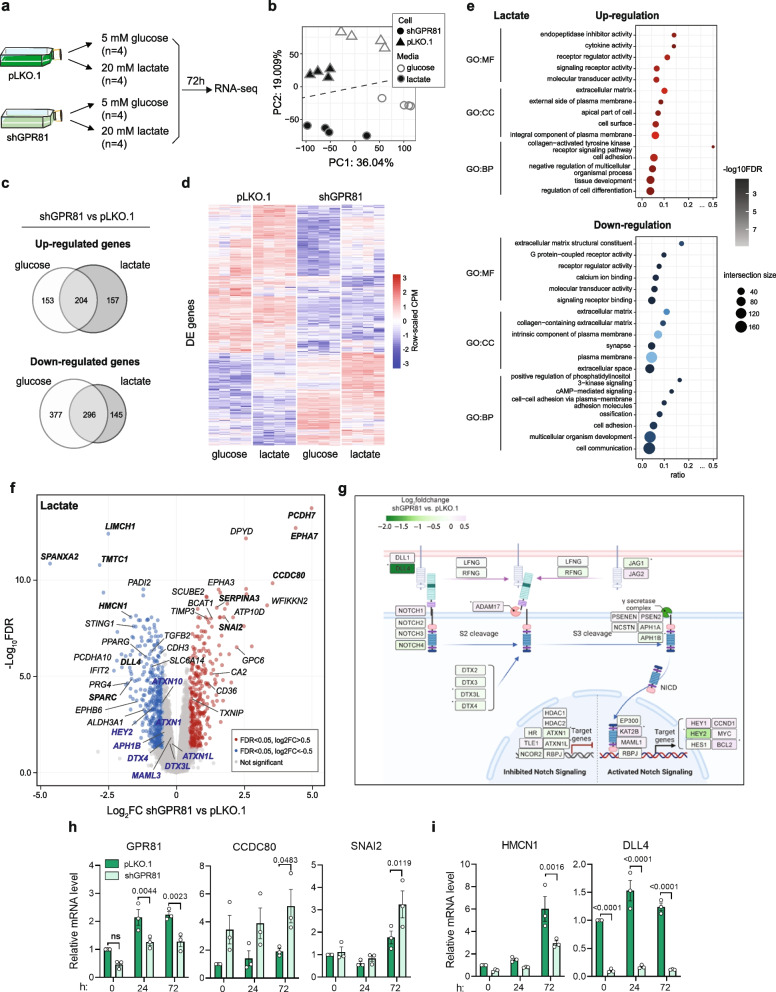
GPR81 KD alters expression of genes regulating cell adhesion and ECM organization. **a** Experimental setup. **b** Principal component (PC) analysis of RNA-seq data. PCs 1 and 2 are shown as X- and Y-axes with % explained variance indicated. Triangles and dots represent RNA-seq libraries, coloured by condition. The dotted line separates shGPR81 and pLKO.1 libraries. **c** Venn diagrams showing overlap of significantly differentially expressed (DE) genes (*FDR* < 0.05 and abs log_2 _fold change) > 0.5, by limma analysis) following shGPR81 and pLKO.1 treatment in lactate and glucose conditions. **d** Heatmap of pLKO.1 and shGPR81 cell gene expression after incubation in glucose- or lactate conditions as indicated. Rows correspond to DE genes. Columns correspond to RNA-seq libraries, grouped first by cell type (pLKO.1 or shGPR81) and then by condition (glucose or lactate). Colours correspond to normalized RNA-seq read counts (CPM) that were subsequently row-scaled. **e** GO analysis of DE genes in the lactate condition, split by up- (upper panel) and down-regulated genes (lower panel). Colour intensity indicates over-representation significance (-log_10_-scaled *FDR*), while dot size indicates intersection size (number of DE genes with a given GO term). GO terms are ordered by class: molecular function (MF), cell compartment (CC) and biological process (BP). **f** Volcano plot based on RNA-seq data analysis of GPR81 KD in lactate environment. Dots correspond to genes, coloured by DE status as defined above. Genes of particular interest are labelled by name. Gene names in bold are discussed specifically in main text. Gene names in blue are Notch pathway genes with *FDR* < 0.05 but absolute log_2 _fold change < 0.5, by limma analysis. See text for details. **g** Expression changes of expressed genes involved in the Notch signaling pathway, color-coded by log_2_foldchange obtained from differential expression analysis between shGPR81 and pLKO.1 samples in lactate condition. Included genes based on KEGG map04330 [46]. Asterisk indicates FDR < 0.05. **h**-**i** qPCR validation of selected upregulated (**h**) and downregulated (**i**) DE genes, shown as mean ± SEM of relative mRNA level normalized to pLKO.1 per time point. Statistics: Two-way ANOVA

To gain further insight into the molecular mechanisms underlying the observed changes in cancer cell adhesion and motility upon GPR81 KD, we next performed whole transcriptome analysis by RNA-seq, of pLKO.1 and shGPR81 MCF-7 cells cultured for 72 h in glucose- or lactate medium, respectively (Fig. 4a). Principal component (PC) analysis could separate both cell type (control and GPR81 KD) and treatment (glucose and lactate) with the first two PCs (Fig. 4b), arguably with more distinct separation between media than between pLKO.1 and shGPR81. Compared to pLKO.1 cells, 357 genes were significantly upregulated (*FDR* < 0.05 and log_2_ fold change > 0.5, by limma analysis) upon GPR81 KD in the glucose-, and 361 in the lactate condition; of these, 204 genes were upregulated in both conditions (Fig. 4c, top). Similarly, 673 genes were significantly downregulated (*FDR* < 0.05 and log_2_ fold change < 0.5, by limma analysis) upon GPR81 KD in the glucose-, and 441 in the lactate condition, and of these, 296 genes were shared (Fig. 4c). Thus, the number of significant differences between control and GPR81 KD cells was greater than between glucose- and lactate conditions. This pattern was also evident when plotting expression levels of all DE genes as a heatmap (Fig. 4d; for all DE genes, see Suppl. Tables 1, 2, 3 and 4).

Based on the striking phenotypical effects of high lactate described above, we focused on DE genes upon GPR81 KD in this condition. Figure 4e shows an overview of overrepresented GO terms for these genes (Suppl. Tables 5 an 6 list all GO term analyses) and Fig. 4f shows a volcano plot of the DE genes. GO terms enriched in the set of genes upregulated upon GPR81 KD were dominated by terms related to Cell-ECM interaction (e.g. Extracellular matrix, Cell adhesion, External side of plasma membrane). As expected, GO terms for the downregulated set included GPCR signaling and downstream pathways (e.g. Positive regulation of PI3K signaling), but also ECM-cell adhesion terms such as Extracellular space, Cell adhesion, and Collagen-containing extracellular matrix. The latter was exemplified by Hemicentin-1 (HMCN1) [47], downregulation of which upon GPR81 KD was confirmed by qPCR (Fig. 4i).

Looking at specific genes rather than overall GO terms, some of the most up-regulated genes upon GPR81 KD included Protocadherin H7 (PCDH7), which is a non-clustered cadherin of the cadherin superfamily [48], the ephrin receptor A7 (EPHA7), coiled-coil domain containing 80 (CCDC80) which is a secreted protein that binds to ECM proteins [49] and SNAI2 (SLUG: discussed below) (Fig. 4f,h). The two latter genes are annotated with most of the ECM and cell adhesion GO terms discussed above, and their gradual upregulation in GPR81 KD cells in lactate medium was confirmed by qPCR (Fig. 4h). The most highly downregulated genes in GPR81 KD cells included Sperm protein associated with the nucleus X-chromosome-A2 (SPANXA2) [50], Transmembrane O-Mannosyltransferase Targeting Cadherins-1 (TMTC1) [51], and LIM and calponin homology domain 1 (LIMCH1), an actin-regulatory protein implicated in cervical cancer development [52] (Fig. 4f).

SNAI2, which as noted above was upregulated upon GPR81 KD, is a negative regulator of signaling by the Notch ligand Delta-Like Ligand 4 (DLL4) [53]. Accordingly, using KEGG pathway analysis, we noted that several elements of Notch signaling were downregulated upon GPR81 KD (Fig. 4g). These included DLL4 itself, strongly reduced expression of which upon GPR81 KD was also confirmed by qPCR (Fig. 4i), the Notch-regulated transcription factor HEY2 (Fig. 4f), and to a lesser extent, other elements of Notch signaling including Jagged-1 (JAG1), the γ-secretase subunit APH1B, Ataxin-1 and -10 (ATXN1, -10), Deltex E3 ubiquitin ligase 4 (DTX4), and Mastermind Like Transcriptional Coactivator 3 (MAML3) (Suppl. Table 7, summarized in Fig. 4g).

To evaluate the relevance of this in breast cancer patients, we studied the expression of the sets of genes most up- and down regulated with GPR81 KD in our study (i.e. expected to de- and increase with increasing GPR81 expression, respectively) in bulk and single cell RNA seq data from human breast cancer patients (Suppl. Figure 5a-b). GPR81 is just one of numerous factors regulating these genes, and the bulk RNA seq data reflect expression in all cell types of the tumor, not just the cancer cells. It is therefore interesting to note that expression of the set of genes upregulated with GPR81 KD in our study decreases with increasing GPR81 expression, consistent with the prediction (Suppl. Figure 5a). This pattern is not seen at low GPR81 levels and the expected upregulation is not seen for the set of GPR81-KD-downregulated genes. To focus on Luminal subtype cancer cells, we analysed five single cell sets of ER positive epithelial cancer cells using the same GPR81-KD gene sets (Suppl. Figure 5b). Interestingly, in 3 of the 5 sets, expression of the KD-downregulated set initially increased with increasing GPR81 expression, while the KD-upregulated set did not, or much less steeply. Furthermore, the gene set positively regulated by GPR81 in our study showed a strong tendency to be the most highly expressed in the tumor tissue (Suppl. Figure 5b). Importantly, correlation analysis using bulk RNA seq data from TCGA furthermore showed that DLL4 mRNA expression correlated with GPR81 expression in human breast cancer tissue (Suppl. Figure 5c). Furthermore, qPCR analysis showed that DLL4 expression in MDA-MB-231 increased in parallel with GPR81 expression with increasing spheroid size (Suppl. Figure 5d, compare with GPR81 expression in Fig. 2d).

Stimulation of MCF-7 p.LKO.1 cells with the synthetic, non-metabolite GPR81 agonist AZ38 generally confirmed this pattern: as expected, SNAI2 and TIMP3, which were upregulated by GPR81 KD, were downregulated by 24 h of AZ38 treatment, and CLDN1 which was downregulated by GPR81 KD, was upregulated by AZ38. SERPINA3 was upregulated by both GPR81 KD and AZ38 treatment, possibly suggesting a difference between loss and inhibition of the protein, which was not further studied here (Suppl. Figure 5e, compare with Fig. 4f,i and Suppl. Tables 3 and 4).

In summary, these results show that mRNA levels of genes involved in regulation of cell adhesion, ECM organization, and Notch signaling are altered upon GPR81 knockdown, at least partly correlating with the expression of these genes in patient breast tumor tissue.

### PCDH7 co-clusters with other GPR81-regulated genes in Ki67-negative cells in GPR81 KD MCF-7 spheroids

**Fig. 5:**
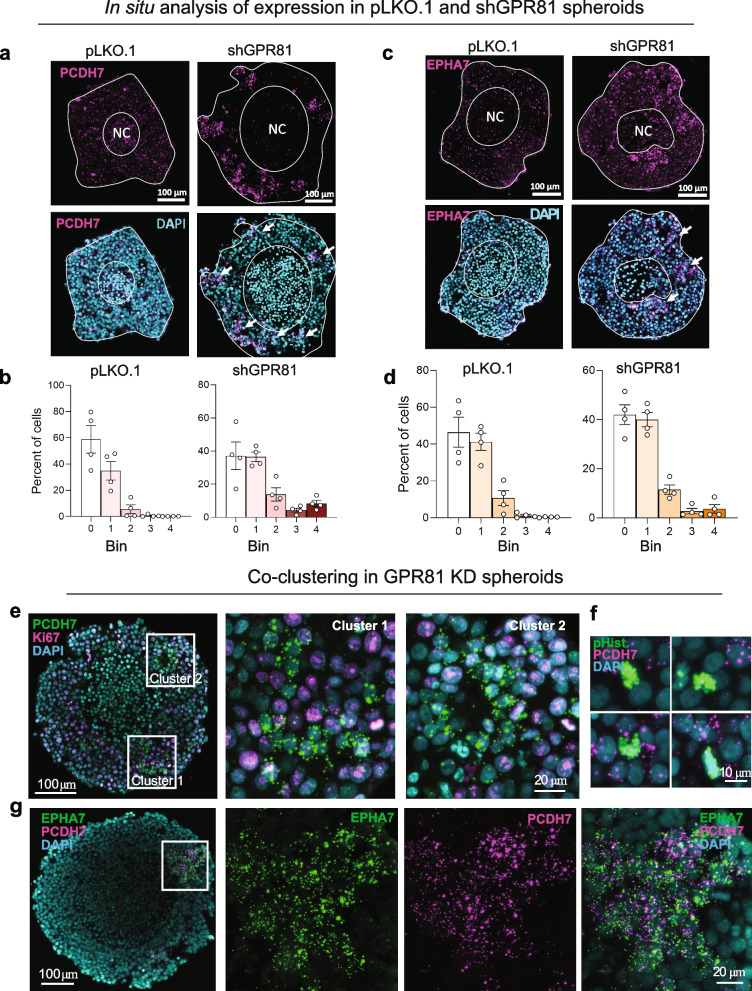
PCDH7 and EPHA7 exhibit clustered upregulation in spheroids upon GPR81 KD. **a**-**d** In situ hybridization of PCDH7 and EPHA7 in pLKO.1 and shGPR81 MCF-7 spheroids. Representative images and corresponding quantification of in situ hybridization of PCDH7 (**a, b**) and EPHA7 (**l, m**) in MCF-7 pLKO.1 and shGPR81 spheroids. **b,d** Quantification of the in situ signal. The Y-axis shows % cells with a given bin distribution (for a detailed description, see Materials and Methods). **e**-**g** High resolution in situ analysis of GPR81 KD (shGPR81) spheroids, detected by RNAscope.. In **e** and **g**, the middle and right panels represent higher magnifications of the boxed regions, illustrating PCDH7-positive cell clusters. **e** Ki67 positive cells (magenta) very rarely exhibit co-expression with PCDH7 (green). **f** Examples illustrating that p-H3 positive cells (green) do not stain for PCDH7 (magenta). **g** PCDH7 (magenta) co-localizes in cell clusters with EPHA7. Representative of 3 biological n per condition

To study the expression pattern of some of the interesting DE genes at a more detailed level, we turned to in situ analysis of MCF-7 spheroids. Firstly, we noted that PCDH7 (Fig. 5a-b) and EPHA7 (Fig. 5c-d) were, surprisingly, upregulated only in a subpopulation of cells in characteristic clusters in the GPR81 KD spheroids, whereas their expression appeared more homogeneous in all non-necrotic cells of the pLKO.1 control spheroids (Fig. 5a,c). Confirming this, the fraction of cell with the highest PCDH7 copy numbers (bin 4), increased from zero in pLKO.1 to 8% in GPR81 KD spheroids (Fig. 5a-b). A similar pattern, albeit a lower overall expression level, was observed for EPHA7 (Fig. 5c-d).

Next, we used high resolution imaging to investigate the striking clustering of PCDH7 and EPHA7 in GPR81 KD spheroids in further detail. Importantly, co-staining for Ki67 as a marker of proliferation revealed that PCDH7 and Ki67 staining was essentially mutually exclusive at the level of individual cells (Fig. 5e). Similarly, cells staining positive for p-H3 as another marker of cell proliferation were also negative for PCDH7 (Fig. 5f). Notably, PCDH7 and EPHA7 staining clearly overlapped, with high co-expression of the two genes in the same cells (Fig. 5g).

Because of the strong indication of altered Wnt signaling upon GPR81 KD, we next examined the spatial expression pattern of DLL4, a Wnt ligand which was strongly downregulated at the mRNA level upon GPR81 KD (see Fig. 4f,i). In contrast to PCDH7 and EPHA7, DLL4 was highly and homogenously expressed in MCF-7 spheroid cells (Suppl. Figure 6a), and quantitative analysis demonstrated a clear reduction in DLL4 expression upon GPR81 KD (Suppl. Figure 6b). Thus DLL4 did not exhibit the striking punctate clustering observed for PCDH7 and EPHA7. We also examined several other GPR81-regulated genes identified in the RNA-seq analysis. Of these, EPHA3 (Suppl. Figure 6c) and TMTC1 (Suppl. Figure 6d), similar to EPHA7, were mainly upregulated in relatively few cells which co-clustered with PCDH7 in the GPR81 KD spheroids. However, this was not true for all upregulated genes. For instance, tissue inhibitor of matrix metalloproteases-3 (TIMP3) was very highly upregulated only in relatively few cells, but these were found as single cells or in small clusters of 2–3 cells which only occasionally overlapped with PCDH7 expressing cells (Suppl. Figure 6e). Among other GPR81-regulated genes relevant to ECM and cell adhesion, Signal Peptide, CUB Domain And EGF Like Domain Containing 2 (SCUBE2) [54] and LIM And Calponin Homology Domains-Containing Protein 1 (LIMCH1) [55] were expressed at low levels and HMCN1, laminin subunit beta 1 (LAMB1) [56] and SNAI2 [53] at very low levels and in all cases exhibiting more homogenous expression patterns in the GPR81 KD spheroids (Suppl. Figure 7).

These findings show that upon 3D spheroid growth of GPR81 KD cells, a number of genes related to ECM and cell-adhesion are highly upregulated in a subpopulation of non-proliferating, clustered MCF-7 cells.

### PCDH7 and EPHA7 proteins are strongly upregulated and cluster in spheroids upon GPR81 KD, but their KD does not restore GPR81-driven migration and invasion

**Fig. 6:**
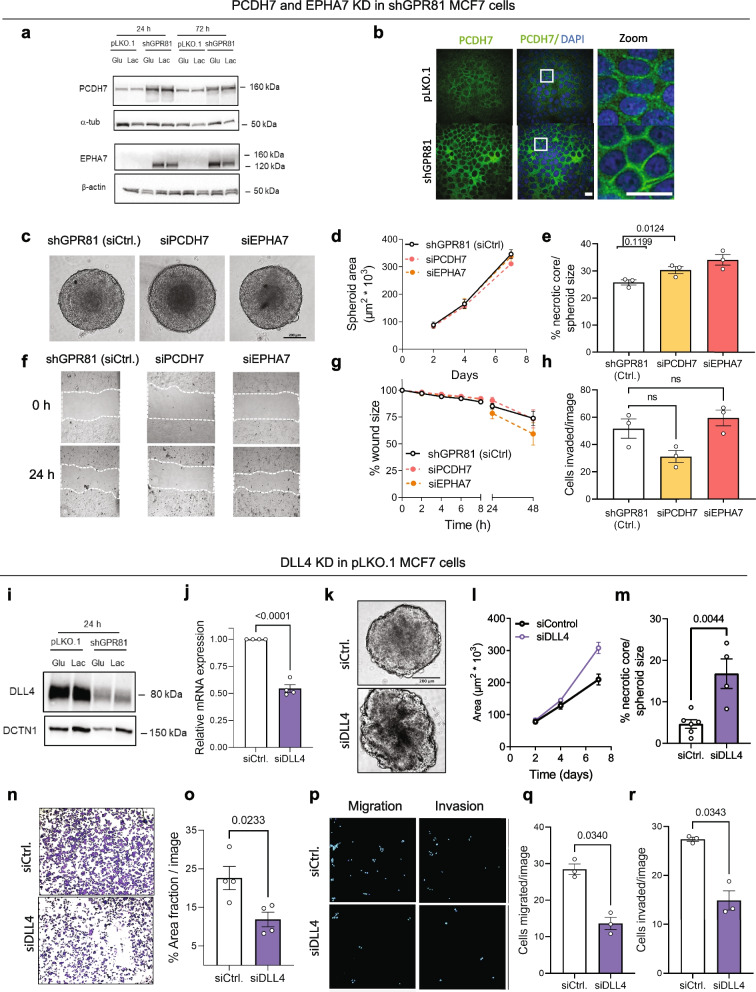
DLL4, but not PCDH7 and EPHA7, contribute to the GPR81-mediated changes in cell survival, migration and invasion. **a** Representative Western blots of PCDH7 and EPHA7 protein levels in pLKO.1 and shGPR81 MCF-7 cells grown in glucose or lactate medium for the time indicated. α-tubulin serves as a loading control for PCDH7 and β-actin for EPHA7. **b** Immunofluorescence analysis of PCDH7 in pLKO.1 and shGPR81 MCF-7 cells grown in lactate medium for 24 h. Scalebar: 20 µm. **c**-**e** Spheroid growth of GPR81 KD MCF-7 cells with/without siRNA-mediated KD of PCDH7 or EPHA7. **c** Representative images, day 7 (*n* = 3). Scalebar: 200 µm. **d** Spheroid area (µm^2^ * 10^3^). **e** Percent necrotic core relative to spheroid size, day 7. **f**-**g** Migration of GPR81 KD MCF-7 cells in lactate medium with/without KD of PCDH7 or EPHA7. **f** Representative images, *n* = 3. Scalebar: 500 µm. **g** Percent wound remaining after 48 h, normalized to control. **h** Boyden chamber invasion assay, quantification, invaded cells/image (*n* = 3). shGPR81 PCDH7 and EPHA7 cells were cultured in lactate medium for 24 h, seeded on matrigel in lactate medium in the upper chamber, and allowed to invade 24 h toward lactate medium with 10% FBS in the lower chamber. **i** DLL4 protein level is decreased by GPR81 KD. pLKO.1 and shGPR81 MCF-7 cells were cultured in glucose or lactate medium for 24 h. Representative Western blot, DCTN1 serves as a loading control (*n* = 3). **j** qPCR validation of siRNA mediated DLL4 KD in MCF-7 cells. **k**-**m** DLL4 KD increases necrotic core size in 3D spheroids of MCF-7 cells. **k** Representative day 7 images (*n* = 3). Scalebar: 200 µm. **l** Average spheroid area (µm^2^ * 10^3^). **m** Percent necrotic core relative to spheroid size on day 7. **n**–**o** Adhesion of MCF-7 cells to matrigel is reduced by DLL4 KD. Cells were incubated for 24 h in lactate medium and seeded on matrigel. After 1 h, nonadherent cells were washed of and cells fixed and stained. **n** Representative images (*n* = 4). **o** Percent area-fraction/image. **p**-**r** DLL4 KD inhibits migration and invasion. Boyden chamber assays of MCF-7 cells with or without siRNA mediated KD of DLL4 or SPARC. MCF-7 cells with/without DLL4 KD were cultured in lactate medium for 24 h, seeded on matrigel in lactate medium in the upper chamber, and allowed to invade 24 h toward lactate medium with 10% FBS in the lower chamber (*n* = 3). **p** Representative images (*n* = 3). **q**-**r** Cells migrated/image (**q**) and cells invaded/image (**r**)

Because of the strong upregulation of PCDH7 and EPHA7 upon GPR81 KD and their striking organization in GPR81 KD MCF-7 spheroids, we next explored the impact of these proteins on MCF-7 cell function. Confirming the RNA-seq data, both proteins were strongly upregulated upon GPR81 KD (Fig. 6a-b). If upregulation of PCDH7 or EPHA7 was essential for the effect of GPR81, their KD in GPR81 KD cells should rescue the WT phenotype. However, KD of PCDH7 or EPHA7 in GPR81 KD cells had no effect on spheroid area (Fig. 6c-d), and EPHA7 KD even increased necrotic core size (Fig. 6c,e), i.e. opposite of what was expected if EPHA7 contributed to the effect of GPR81 KD on growth and necrosis. Furthermore, KD of PCDH7 or EPHA7 in GPR81 KD cells had no effect on cell migration (Fig. 6f-g) or invasion (Fig. 6h). These results show that upregulation of PCDH7 or EPHA7 cannot account for the reduced migration and invasion of MCF-7 cells upon GPR81 KD.

### The Notch ligand DLL4 is important for GPR81-driven migration and invasion of MCF-7 cells

Among the genes downregulated upon GPR81 KD, we choose DLL4 for further analysis in light of its potent downregulation and the importance of DLL4-Notch signaling in cancer [57, 58]. The DLL4 protein level was strongly downregulated upon GPR81 KD (Fig. 6i). Similar to GPR81 KD, KD of DLL4 in WT MCF-7 cells increased spheroid area slightly and almost tripled the size of the necrotic core (Fig. 6j-m), although it did not reproduce the tighter spheroid organization seen upon GPR81 KD (compare Fig. 6k and Fig. 2j). Importantly, DLL4 KD reduced MCF-7 cell adhesion on matrigel by ~ 60% (Fig. 6n-o) and inhibited cell migration by ~ 60% and invasion by ~ 50% (Fig. 6p-r).

These results show that DLL4 is strongly positively regulated by GPR81 in MCF-7 cells and is likely to contribute to the role of GPR81 in regulation of MCF-7 cell growth, migration and invasion.

## Discussion

Recent work has assigned the lactate receptor GPR81 roles in cancer development [9, 17, 18, 43]. The mechanisms remain essentially unclear, although GPR81 has been suggested to regulate cancer cell metabolism [18, 43, 59], angiogenesis [17], and anti-tumor immunity [9, 24, 37]. Here, we show that GPR81 regulates pathways controlling ECM composition, cell adhesion, and signaling in MCF-7 breast cancer cells as a model of Luminal A breast cancers. We further show that this plays a key role in the effects of the receptor on cancer cell 3D growth and invasiveness, likely in part through regulation of Notch ligand DLL4. This is particularly relevant as GPR81 expression correlates with poor survival in Luminal A breast cancers.

We find that albeit highly variable, GPR81 expression is generally upregulated in patient tumor tissue and cancer cells, confirming earlier reports [9, 18, 43]. We show that GPR81 expression varies between breast cancer subtypes, with Luminal A tumors exhibiting the highest, and TNBC tumors the lowest, expression. Using in situ staining of cancer patient TMAs, we show that GPR81 expression exhibits extensive inter- and intra-tumor heterogeneity. We confirm that GPR81 expression is stimulated by lactate [37], which likely at least in part explains our new finding that expression of the receptor increases as 3D spheroids grow, a condition shown to mimic TME conditions including lactate accumulation [38]. We suggest that the heterogeneity of GPR81 expression in patient tumors is driven in part by heterogeneity of TME conditions such as hypoxia and acidosis, and consequent differenes in lactate accumulation. In the future, tumor profiling and single cell expression analysis should ascertain which cell types in the tumors, beyond the cancer cells, express the receptor. This is particularly important as GPR81 expression in tumor-associated immune cells may contribute to immune evasion [9].

Our orthotopic xenograft model confirmed earlier findings that GPR81 KD reduces mammary tumor growth [17, 43] and combining this with 3D culture allowed us to reveal that proliferation was unaltered or even increased in peripheral regions of spheroids. Importantly, the necrotic core of GPR81 KD spheroids nearly doubled in size and spheroid morphology tightened, strongly indicative of cell–cell adhesion changes. Accordingly, we showed that cell adhesion, migration and invasion were attenuated by GPR81 KD in both luminal A and TNBC breast cancer cell models. This is completely consistent with previous work demonstrating a role for GPR81 in supporting these processes in breast cancer models [17, 43]. Accordingly, genes regulated upon GPR81 KD in MCF-7 cells were strongly dominated by GO terms related to ECM composition, cell adhesion, and signaling. For instance, the most highly and significantly upregulated gene upon GPR81 KD was Protocadherin H7 (PCDH7) which belongs to the cadherin superfamily of cell–cell adhesion proteins [48] and is assigned context-dependent roles in various cancers [60]. Also EPHA3 and –7 were upregulated by GPR81 KD. Ephrin receptors constitute the largest family of receptor tyrosine kinases, and while they also play context-dependent roles, their function favors cell–cell adhesion in epithelia and hence is often anti-tumorigenic [61]. Nevertheless, the effect of GPR81 KD was not reverted by KD of PCDH7 or EPHA7 in MCF-7 cells. While not addressed here, such an effect might be revealed in a more complex TME, where ephrins (Ephrin receptor ligands), are secreted by other cells, limiting tumor expansion and invasion [61]. Intriguingly however, PCDH7, EPHA3, -7, and TMTC1 were all highly co-upregulated in a population of Ki67- and pH3-negative, non-proliferating clusters of cells in GPR81 KD spheroids. The GPR81 KD cells are a stable line under continuous selection pressure, and the pattern was not observed for all DE genes, thus, the cluster pattern cannot readily be explained by artifacts or cell-to-cell differences in GPR81 KD. In congruence, environment-driven heterogeneity of monoclonal cell lines in spheroids was recently demonstrated [62]. We therefore propose that the co-clustering reflects local microenvironmental forces driving formation of cell clusters with specific properties and signaling interactions among the clustered cells.

Also genes downregulated by GPR81 KD, i.e. positively regulated by GPR81, were dominated by GO terms related to adhesion, ECM, and cytoskeleton, consistent with the apparent increase in cell–cell adhesion and inhibition of cell–matrix adhesion, migration and invasion upon GPR81 KD. On the other hand, GPR81 may not activate a fully classical EMT program, for instance expression of the EMT transcription factor SNAI2 was increased upon GPR81 KD. In contrast, we noted a striking downregulation of Notch signaling components upon GPR81 KD. We chose to focus on DLL4, which was dramatically downregulated also at the protein level. DLL4 signaling is often associated with regulation of tumor angiogenesis [63], but also serves cancer cell-autonomous roles in tumor growth and metastasis [58]. The strong regulation of Notch signaling by GPR81 is fully in line with its known regulation by microenvironmental factors and ECM interactions [64]. As such, it is interesting to note that SNAI2, which was upregulated upon GPR81 KD, is a negative regulator of DLL4-Notch signaling, acting by binding directly to the DLL4 promoter and likely acting as a transcriptional repressor [53]. A working hypothesis and data summary is shown in Suppl. Figure 8. Corroborating the relevance of these results to the patient setting, DLL4 expression also correlated positively with GPR81 expression in human breast tumors and spheroids (Suppl. Figure 5c-d). The precise pathways through which GPR81 regulates such a plethora of ECM components and -regulators, and how this is linked to DLL4-Notch pathway signaling, remain to be unraveled. In normal cells, GPR81 signals almost exclusively via a G_i_-dependent decrease in cAMP to inhibit lipolysis [15, 16] yet whether this is also the case in cancer cells is an open question.

In summary, the lactate receptor GPR81 is upregulated in many different types of human cancers originating from tissues where GPR81 is normally not detectably expressed. In breast cancer cells, GPR81 is upregulated by lactate and in spheroid culture and favors cancer cell survival, migration, invasion and in vivo tumor growth. GPR81 depletion in MCF-7 cells alters expression of a plethora of genes associated mainly with ECM remodeling, adhesion, and signaling. PCDH7 and EPHA7 were upregulated in GPR81 KD MCF-7 spheroids, co-clustering with other ECM-related gene products. Notch signaling components, and in particular the Notch ligand DLL4, were dependent on GPR81 for expression, and DLL4 KD inhibited MCF-7 cell growth, migration and invasion in a manner similar to that of GPR81 KD (Suppl. Figure 8). We conclude that TME-driven GPR81 upregulation supports Luminal A breast cancer cell aggressiveness at least in part via DLL4. Our findings reveal a new GPR81-driven mechanism in breast cancer and substantiate GPR81 as a promising treatment target.

### Supplementary Information

**Additional file 1.**

## Data Availability

RNA-sequencing data from this study has been deposited in GEO database under accession number GSE203441. Additional information and raw data are available upon reasonable request to the corresponding author.

## Footnotes

1 When de Castro Abrantes et al*.* tested nine commercially available GPR81 antisera in IHC and Western blot analysis, all gave similar results in samples from WT and GPR81 deficient mice. We have observed similar unspecific effects for several of these antisera in GPR81 transfected and -untransfected cells.
