# Supplementary material for: Lactate receptor GPR81 drives breast cancer growth and invasiveness through regulation of ECM properties and Notch ligand DLL4
Source: BMC Cancer. 2023 Nov 22;23:1136. doi: 10.1186/s12885-023-11631-6 (PMC10666402; doi:10.1186/s12885-023-11631-6)
Supplement: Supplementary file 1 — Additional file 1. [file 12885_2023_11631_MOESM1_ESM.zip › Lundo et al_Suppl materials_figs_tables_2023-09-24_final.pdf]

SUPPLEMENTARY MATERIALS

for

Lactate receptor GPR81 drives breast cancer growth and invasiveness through regulation of ECM properties and Notch ligand DLL4

Kathrine Lundø[1], Oksana Dmytriyeva[1], Louise Spøhr[2], Eliana Goncalves-Alves[2], Jiayi Yao[3,4], Laia P. Blasco[4], Mette Trauelsen[1], Muthulakshmi Ponniah[2], Marc Severin[2], Albin Sandelin[3,4], Marie Kveiborg[4], Thue W. Schwartz[1,*], Stine F. Pedersen[2*]

[1]Novo Nordisk Foundation Center for Basic Metabolic Research, Faculty of Health and Medical Sciences, The Panum Institute, DK-2200 Copenhagen, Denmark; [2]Section for Cell Biology and Physiology, Department of Biology, Faculty of Science, University of Copenhagen, DK-2100 Copenhagen, Denmark; [3]The Bioinformatics Centre, Department of Biology, Faculty of Science, University of Copenhagen; [4]Biotech Research and Innovation Centre, University of Copenhagen, DK2200 Copenhagen, Denmark

Short title: GPR81-mediated signalling drives breast cancer progression

* Correspondence:

Thue W. Schwartz: tws@sund.ku.dk

Stine F. Pedersen: sfpedersen@bio.ku.dk

THIS SUPPLEMENT CONTAINS:

- Supplementary Methods
- Supplementary Figures 1-7
- Legends for supplementary tables 1-7
- Supplementary tables 8-11

SUPPLEMENTARY METHODS

GPR81 mRNA expression in patient tissues

mRNA expression of GPR81 (HCAR1) in patient tumor tissue and normal control tissue was analyzed using the Gene Expression Profiling Interactive Analysis (GEPIA) server (http://gepia.cancer-pku.cn/) [1]. Tumor tissue and available normal control data are from The Cancer Genome Atlas (TCGA): https://www.cancer.gov/tcga [2]. To obtain a balanced number of tumor- and control samples, the GEPIA server includes additional normal control values from The Genotype-Tissue Expression project (GTEx) [3] project after re-computing both TCGA and GTEx gene expression data from raw RNA-Seq data by the UCSC Xena project (http://xena.ucsc.edu/) minimize differences resulting from distinct sources, as described in [1].

Cell lines and cell culture

MCF-7 (ATCC, #HTB-22) and MDA-MB-231 (ATCC, HTB-26) were grown in DMEM supplemented with 1% non-essential amino acids (NEAA) (#M7145, Sigma-Aldrich), 1% Penicillin/Streptomycin (pen/strep) (#069, SSC, University of Copenhagen) 2 mM glutamine and 10% FBS. T47D cells (ATCC, HTB-133) were grown in RPMI 1640 (#075, SSC, University of Copenhagen) supplemented with 10 % FBS and 0.5 % Insulin-Transferrin-Selenium (41400-045, Gibco) and 1% pen/strep. SKBr-3 cells (ATCC-HTB-30) were grown in McCoy's 5a Modified Medium (#M9309, Sigma) supplemented with 10% FBS and 1% pen/strep. BxPC-3 (ATCC, # CRL-1687), Capan-2 (kindly gifted from Luis Arnes Perez, BRIC, University of Copenhagen), SKOV-3 (kindly gifted from Tuula Kallunki, Danish Cancer Society Research Center) and RT4 cells (#91091914, Sigma) were grown in RPMI-1640 (#075, SSC, University of Copenhagen) supplemented with 1% pen/strep, 2 mM glutamine and 10% FBS. LoVo (#CCL-229, ATCC) were grown in Ham's F12 (#032, SSC, University of Copenhagen) supplemented with 1% pen/strep, 2 mM glutamine and 10% FBS. MCF10A cells were grown in a 1:1 mix of DMEM (#41966, Gibco) and Ham's F12 nutrient mixture medium (#N6658, Sigma) supplemented with 1% pen/strep, 5% FBS, 20 ng/ml recombinant human

epidermal growth factor (#E9644, Sigma), 0.25 ng/mL hydrocortisone (#H0888, Sigma), and 10 µg/ml bovine insulin-transferrin-selenium (#41400-045, Gibco). Panc-1 cells were grown in DMEM (#32430-027, Gibco) supplemented with 1% pen/strep and 10% FBS.

siRNA mediated knockdown

siRNA information is found in Suppl. Table 9. siRNA transfections were performed according to manufacturer's instructions. GPR81 RNA oligonucleotide (siGPR81, 5'-GAAGAGAUGCCAAUUUCGA-3 (fw), 5-'UCGAAAUUGGCAUCUCUUC-3' (rv) and siControl, MISSION® siRNA Universal Negative Control #1 were used (#SIC001, Sigma Aldrich). siRNA to a final concentration of 0.5 ng/ml was mixed with Lipofectamine 3000 (#L3000015, ThermoFisher) according to manufacturer's instructions. Oligomer-Lipofectamine complexes were then gently added to the cells. Oligomer-Lipofectamine complexes were gently added to the cells and left to incubate for 48 h before assays at 37°C, 5% CO_2.

Agarose fixation and paraffin embedding of spheroids

Spheroids were isolated and fixed in 4% PFA for 24 h prior to embedding. An agarose gel was prepared using Bacto™ Agar (#214050, BD) diluted in MiliQ water and heated until fully melted. The agarose was put on Superfrost Ultra Plus Object Glasses (#J380AMNZ, Thermo Fisher) in drops and 15-20 spheroids were then injected into the center of the drop and left to solidify. The spheres were put into cassettes and submerged into 96% ethanol for 30 min, 99% ethanol for 2 x 30 min. Subsequently, they were submerged in xylene for 2 x 30 min before being embedded in paraffin.

In situ hybridization and immunohistochemistry

Paraffin-embedded spheroids were cut into 5 µm thin cross-sections using a Microtom HM200 and mounted onto Superfrost slides. The sections were baked at 60°C for 60 min, deparaffinized and rehydrated. Slides were air-dried at room temperature before continuing with a hydrogen peroxide pre-treatment for 10 min and target retrieval by boiling in target retrieval reagent for 5 min. Protease

plus (#322330, Advanced Cell Diagnostics) was added for tissue permeabilization and slides were incubated for 15 min at 40°C. Then tissue was hybridized with choosen probes (Suppl. Table 11) using Multiplex Fluorescent detection reagents v2 (#323110, Advanced Cell Diagnostics) according to the manufacturers protocol. Probe signals were developed by incubation with Opal reagents (Akoya Bioscience). The slides were incubated with blocking buffer (5% donkey serum in PBS) at room temperature for 45 min. Primary antibodies were added and slides were incubated overnight at 4°C. Slides were washed in 1x PBS five times before adding secondary antibodies (1:800 in 5% donkey serum). Slides were incubated for 1 h at room temperature before washing in PBS, and mounted with ProLong™ Gold Antifade Mountant with DAPI (#P36935, Invitrogen). Images were acquired using Zen Pro 3.0 Software connected to a Zeiss Axiocam 702 monochrome microscope camera using a 20X/0.8 NA objective. Expression of chosen markers was evaluated using the HALO image analysis platform which detects intensity of expression in each cell. Cells were divided into 5 bins (0 to 4), where bin 0 contain cells with no detectable expression, bin 1 contains cells with 1-3 dots, bin 2, cells with 4-9 dots, bin 3, cells with 10-15 dots, and bin 4, cells with more than 15 dots.

Cell viability assay

The effect of GPR81 KD on cell viability was determined using CellTiter-Glo Luminescent Cell Viability Assay (#G7570, Promega) according to manufacturer's instructions. Cells were grown under normal conditions overnight and the medium was changed to either 20 mM lactate medium (0 mM glucose, 2% FBS, 1% NEEA) or 5 mM glucose medium (0 mM lactate, 2% FBS, 1% NEEA) and incubated for 72 h before assessing luminescence (RLU).

Western blotting

Cells were lysed in 95°C SDS lysis buffer (0.1 M Tris-HCl, 0.1 M pH 7.5, 1% SDS, 1mM Na_3VO_4 and Complete™ protease inhibitor (#11836153001, Roche). Lysates were homogenized by sonication (PowerMED), centrifuged (Micromax RF, Thermo) for 5 min at 20,000 × g at 4°C, and protein concentrations determined using the DC Protein assay kit (#500-0113, #500-0114, #500-0115,

BioRad). Samples were normalised with ddH_2O and mixed with NuPAGE LDS 4x sample buffer (#NP0007, Invitrogen) and dithiothreitol (DTT). Equal amounts of protein were separated by SDS-PAGE using Criterion 10% Tris gels (Bio-Rad) and Tris/Glycine/SDS running buffer (#161-0732, BioRad), and BenchMark protein ladder (#10747-012, Invitrogen). Proteins were transferred using the Trans-Blot Turbo transfer system (BioRad) to Trans-Blot Turbo 0.2 µm nitrocellulose membranes (#170-4159, BioRad). Membranes were stained with Ponceau S (#P7170-1L, Sigma-Aldrich), blocked for 1 h at 37°C in 5% nonfat dry milk in TBST (0.01 M Tris/HCl, 0.15 M NaCl, 0.1% Tween 20, pH 7.4). Membranes were incubated with primary antibodies (Suppl. Table 10) diluted in 5% nonfat dry milk in TBST overnight at 4°C, and with horseradish peroxidase (HRP) conjugated secondary antibodies in 5% nonfat dry milk in TBST for 1 h at room temperature. Bands were developed by enhanced chemiluminescent (ECL) substrate (#32106, Pierce) or SignalFire (#6883, Cell Signaling) and visualized with Fusion Fx (Vilber Lourmat). Densitometric analyses were carried out using ImageJ.

GPR81 overexpression

Transient transfection of a Flag-tagged GPR81 into MDA-MB-231 cells was carried out using a pCMV-GPR81-Tag2B plasmid and Lipofectamine 3000 (Invitrogen, #L3000015), following the manufacturer's protocol. Spheroids were prepared 48 h post-transfection. Overexpression was confirmed by qPCR analysis.

Analysis of the expression of up- or down-regulated genes following HCAR1 depletion in NN cells and HCAR expression in bulk-seq TCGA RNA-seq data

Pre-normalized TCGA (FKPM) breast cancer RNA-seq data was downloaded from https://xenabrowser.net/datapages/?dataset=TCGA-BRCA.htseq_fpkm.tsv&host=https%3A%2F[%E2%80%A6].net&removeHub=https%3A%2F%2Fxena.treehouse.gi.ucsc.edu%3A443. From this data, we selected samples from luminal A subtype using the associated pam50 classification and from those we selected genes that were upregulated or downregulated by HCAR1 depletion from our data (Table S3 and S4), but using a |log2FC| threshold of 1. For each patient sample, we calculated the mean expression in the up- or

downregulated set. We then plotted this value vs the HCAR1 expression from the same sample, but binned these values into 10 bins. Thus, each HCAR1 expression bin had a number of gene expression means, whose distributions were visualized as boxplots

Analysis of the expression of up- or down-regulated genes following HCAR1 depletion in MCF-7 cells and HCAR expression in single-seq RNA-seq data

We used preprocessed single cell data compiled by [4] and focused on breast cancer single cell data from [5], comprising 45 samples and 305,224 cells. We used Seurat to process data: specifically, we normalised and scaled the expression data using NormalizeData, identified the 15000 most variable feature using FindVariableFeatures and then scaled the data using ScaleData. This was done independently for the 5 data groups in [5]. As above, we then calculated the average (scaled) expression of genes that were upregulated or downregulated by HCAR1 depletion from our data (Table S3 and S4), but using a |log2FC| threshold of 1, for each cell. These sets are already annotated by cell type by the authors, and we focused on epithelia-labelled cells only, and only plotted samples where HCAR1 expression showed variation (e.g, is not only non detected).

GPR81 stimulation with AZ38

To compare the effect of GPR81 KD to that of pharmacological stimulation, we treated MCF7 PLKO.1 cells with the synthetic non-metabolite GPR81 agonist, AZ38 and assessed expression of key genes by GPR81 KD. MCF7 PLKO.1 cells were grown in Optimem (#51985-026), 1% FBS, 1% NEAA, 1% P/S and were stimulated with 1 μM AZ38 for 24 h before qPCR analysis as above, with GAPDH as housekeeping gene.

References

1. Tang Z, Li C, Kang B, Gao G, Li C, Zhang Z: GEPIA: a web server for cancer and normal gene expression profiling and interactive analyses. *Nucleic Acids Res* 2017, 45:W98-W102.
2. McLendon R, Friedman A, Bigner D, Van Meir EG, Brat DJ, M. Mastrogianakis G, Olson JJ, Mikkelsen T, Lehman N, Aldape K *et al*: Comprehensive genomic characterization defines human glioblastoma genes and core pathways. *Nature* 2008, 455(7216):1061-1068.
3. The Genotype-Tissue Expression (GTEx) project. *Nat Genet* 2013, 45(6):580-585.

4. Gavish A, Tyler M, Greenwald AC, Hoefflin R, Simkin D, Tschernichovsky R, Galili Darnell N, Somech E, Barbolin C, Antman T *et al*: Hallmarks of transcriptional intratumour heterogeneity across a thousand tumours. *Nature* 2023, 618(7965):598-606.
5. Pal B, Chen Y, Vaillant F, Capaldo BD, Joyce R, Song X, Bryant VL, Penington JS, Di Stefano L, Tubau Ribera N *et al*: A single-cell RNA expression atlas of normal, preneoplastic and tumorigenic states in the human breast. *The EMBO Journal* 2021, 40(11):e107333.
6. Consortium GT: The GTEx Consortium atlas of genetic regulatory effects across human tissues. *Science* 2020, 369(6509):1318-1330.
7. Cancer Genome Atlas Research N, Weinstein JN, Collisson EA, Mills GB, Shaw KR, Ozenberger BA, Ellrott K, Shmulevich I, Sander C, Stuart JM: The Cancer Genome Atlas Pan-Cancer analysis project. *Nat Genet* 2013, 45(10):1113-1120.

SUPPLEMENTARY FIGURES

Suppl. Figure 1

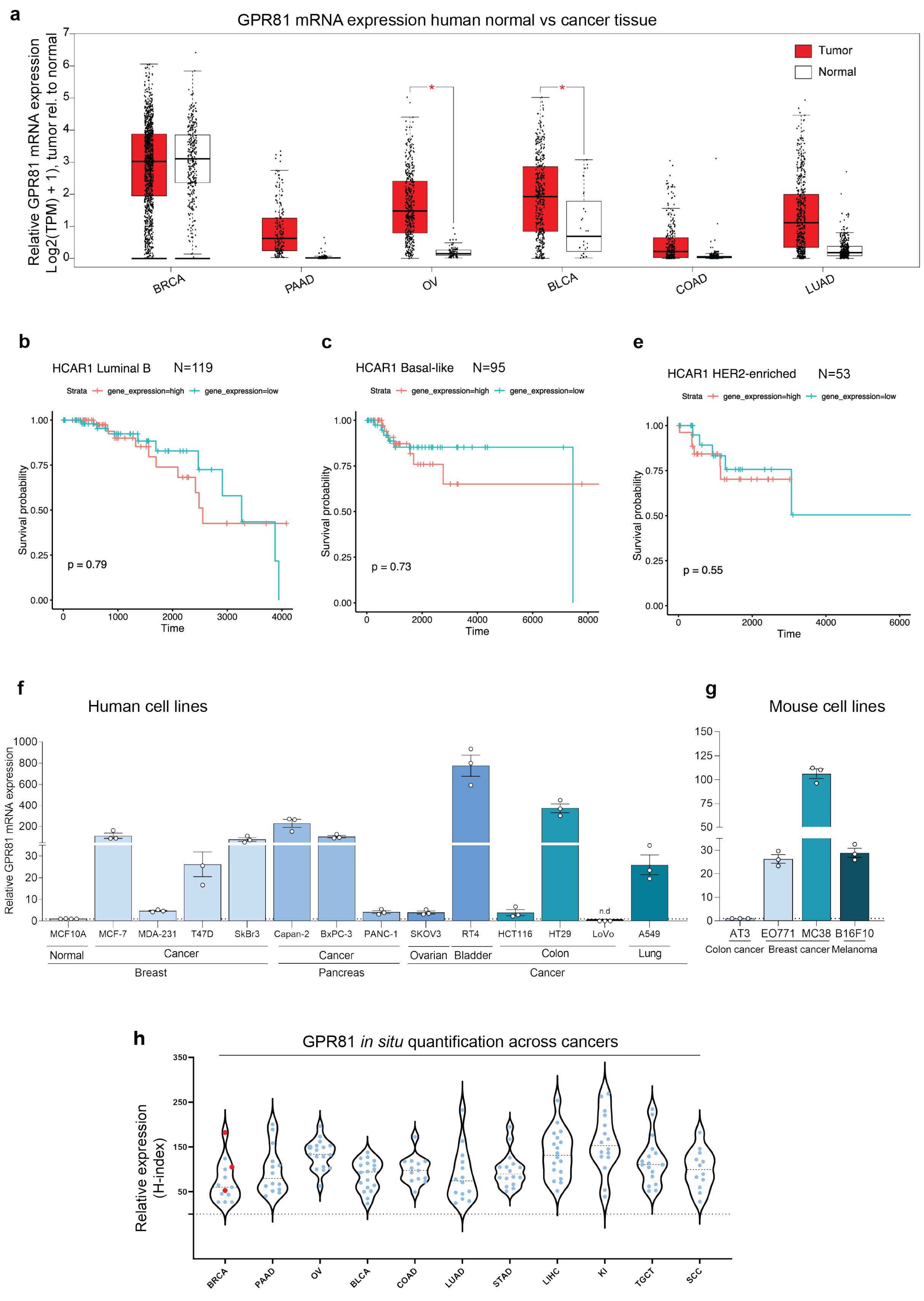

Supplementary Figure 1. GPR81 expression in patient tumors and cell lines, and correlation with patient survival.

a. mRNA expression of GPR81 was analyzed using the Gene Expression Profiling Interactive Analysis (GEPIA) server (http://gepia.cancer-pku.cn/). To ensure balanced numbers of tumor and control samples, GEPIA analysis is based on data from The Cancer Genome Atlas (TCGA) (https://www.cancer.gov/tcga) [6, 7] plus additional normal controls from The Genotype-Tissue Expression (GTEx) project (The Genotype-Tissue Expression (GTEx) project, 2013), recomputed using a common platform (Tang et al., 2017). Y-axis: log2 (transcript count per million (TPM) +1) of tumor relative to normal expression levels. Colored boxes, tumor; open boxes, control. BRCA: Breast invasive carcinoma, PAAD: Pancreatic adenocarcinoma, OV: Ovarian serous cystadenocarcinoma, BLCA: Bladder Urothelial Carcinoma, COAD: Colon adenocarcinoma, LUAD: Lung adenocarcinoma, STAD: Stomach adenocarcinoma, LIHC: Liver hepatocellular carcinoma, KI: Kidney cell carcinoma, TGCT: Testicular Germ Cell Tumors, SCC: Squamous cell carcinoma.

b-c. Kaplan-Meier survival analysis of breast cancer patient data as a function of high or low GPR81 expression. Data for Luminal B, Basal-like, and HER2-enriched tumors are shown. **f-g.** qPCR analysis of relative GPR81 mRNA expression in human (**f**) and murine (**g**) cancer cell lines (n=3 independent replicates per cell line, indicated by dots). Human breast cancer data are also shown in Fig. 1 and are only shown here for comparison. **h.** RNAscope analysis of GPR81 expression across cancers. Y-axis: H-index, a measure of GPR81 expression and heterogeneity. Cells were grouped into 5 bins (groups) based on the number of dots per cell. Each sample was evaluated for the % cells in each bin. The H-index was calculated by totalling % cells in each bin, according to a weighted equation where bin 0 corresponds to 0, bin 1 to 1, etc (see Materials and Methods). Each dot corresponds to a biopsy from single patient. Human breast cancer data are also shown in Fig. 1h and are only shown here for comparison.

Suppl. Fig. 2

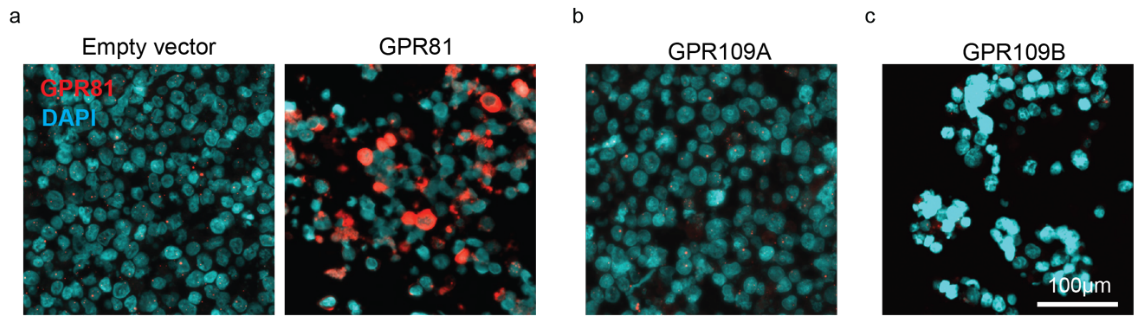

Supplementary Figure 2. Specificity of the GPR81 in situ hybridization probe.

RNAscope analysis of GPR81 in HEK293 cells in 2D culture. Cells were transfected with empty vector, GPR81 (a), GPR109A (b), or GPR109B (c). The experiment demonstrates that probe specifically binds to GPR81 mRNA, but not to GPR109A or GPR109B.

Suppl. Fig. 3

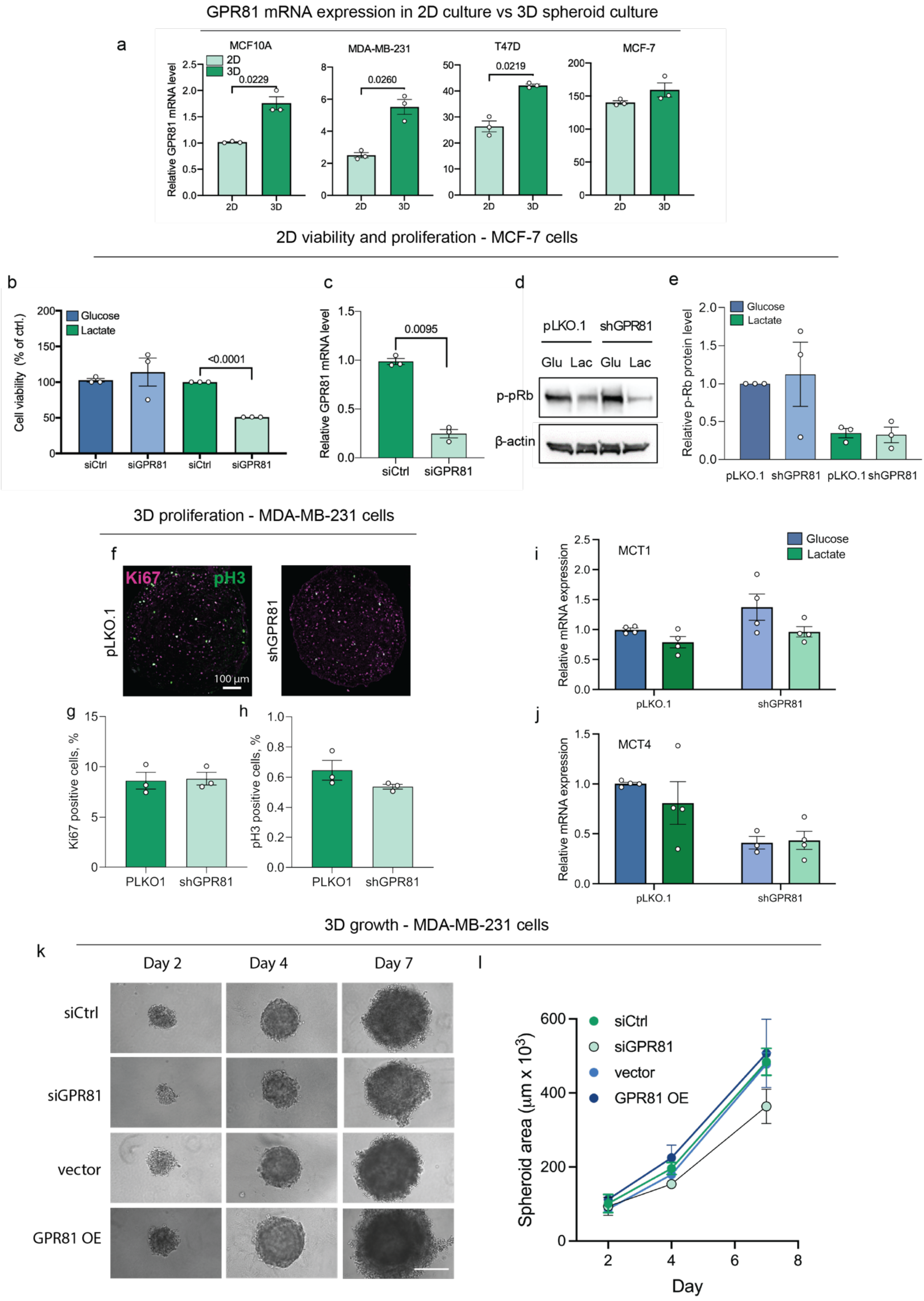

GPR81 mRNA expression in 2D culture vs 3D spheroid culture
a
MCF10A
MDA-MB-231
T47D
MCF-7
2D
3D
Relative GPR81 mRNA level
0.0229
0.0260
0.0219
2D viability and proliferation - MCF-7 cells
b
c
d
e
Glucose
Lactate
Cell viability (% of ctrl.)
siCtrl
siGPR81
<0.0001
Relative GPR81 mRNA level
0.0095
pLKO.1
shGPR81
Glu
Lac
p-pRb
β-actin
Relative p-Rb protein level
3D proliferation - MDA-MB-231 cells
f
Ki67
pH3
100 µm
g
h
Ki67 positive cells, %
pH3 positive cells, %
PLKO1
i
j
MCT1
MCT4
Relative mRNA expression
3D growth - MDA-MB-231 cells
k
Day 2
Day 4
Day 7
vector
GPR81 OE
l
Spheroid area (µm x 10³)
Day

Supplementary Figure 3. GPR81 is upregulated by 3D spheroid growth

a. qPCR analysis of GPR81 mRNA levels in 2D vs day 7 3D cultures of normal (MCF10A) and breast cancer (MDA-MB-231, T47D, MCF-7) cells (n=3). **b-c.** GPR81 was transiently knocked down in MCF-7 cells, and 24 h later, cells were cultured in glucose or lactate medium for 72 h before assessing cell viability, presented as % cell viability compared to control (siCtrl) (**b**, n=3) and siRNA KD efficiency (**c**, n=3). **d-e.** Representative Western Blot and quantification of p-pRB on pLKO.1 and shGPR81 MCF-7 cells incubated in glucose or lactate medium for 24 h. β-actin serves as a loading control and data is presented as relative p-pRB protein level (n=3). **f-h.** Representative images and corresponding quantification of MDA-MB-231 pLKO.1 and GPR81 KD (shGPR81) spheroids stained for Ki67 and phospho-Histone H3 (pH3). Graphs show % live cell positive for Ki67 (g) or pH3 (h). **i-j.** mRNA expression of MCT1 and MCT4 in MCF-7 cells with and without stable GPR81 KD, after 24 h in glucose- or lactate medium. **k-l.** Spheroid growth of MDA-MB-231 cells with either siRNA-mediated GPR81 KD, overexpression of FLAG-tagged GPR81, or the respective controls. k, representative images, l, quantification of spheroid area (n=3).

Suppl. Fig. 4

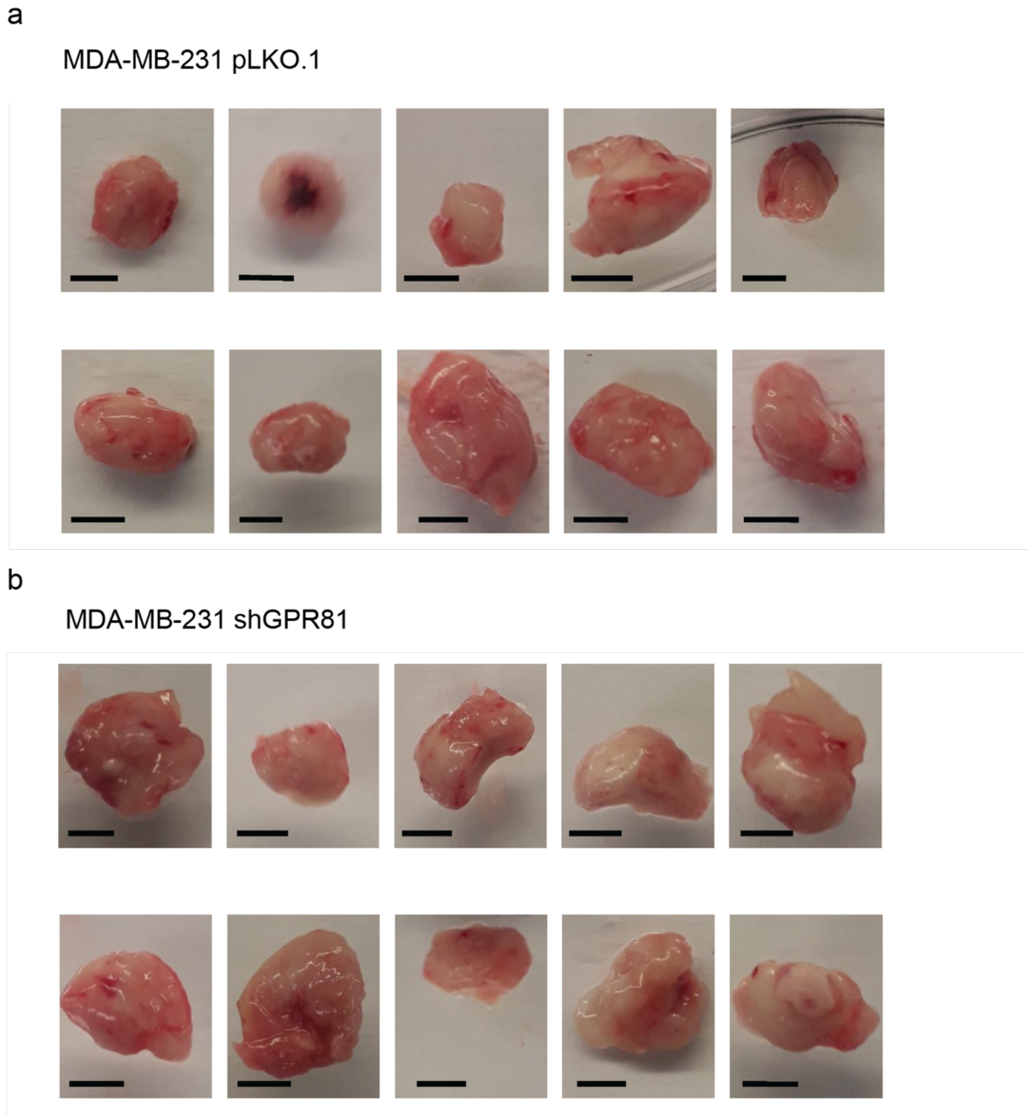

Supplementary figure 4. Orthotopic MDA-MB-231 cell tumors

Morphology of (**a**) MDA-MB-231 pLKO.1 and (**b**) shGPR81 orthotopic xenograft tumors. Tumors were excised when they reached 550-600 mm^3, unless mice were terminated early for ethical reasons. Scale bar: 5 mm.

Suppl. Fig 5

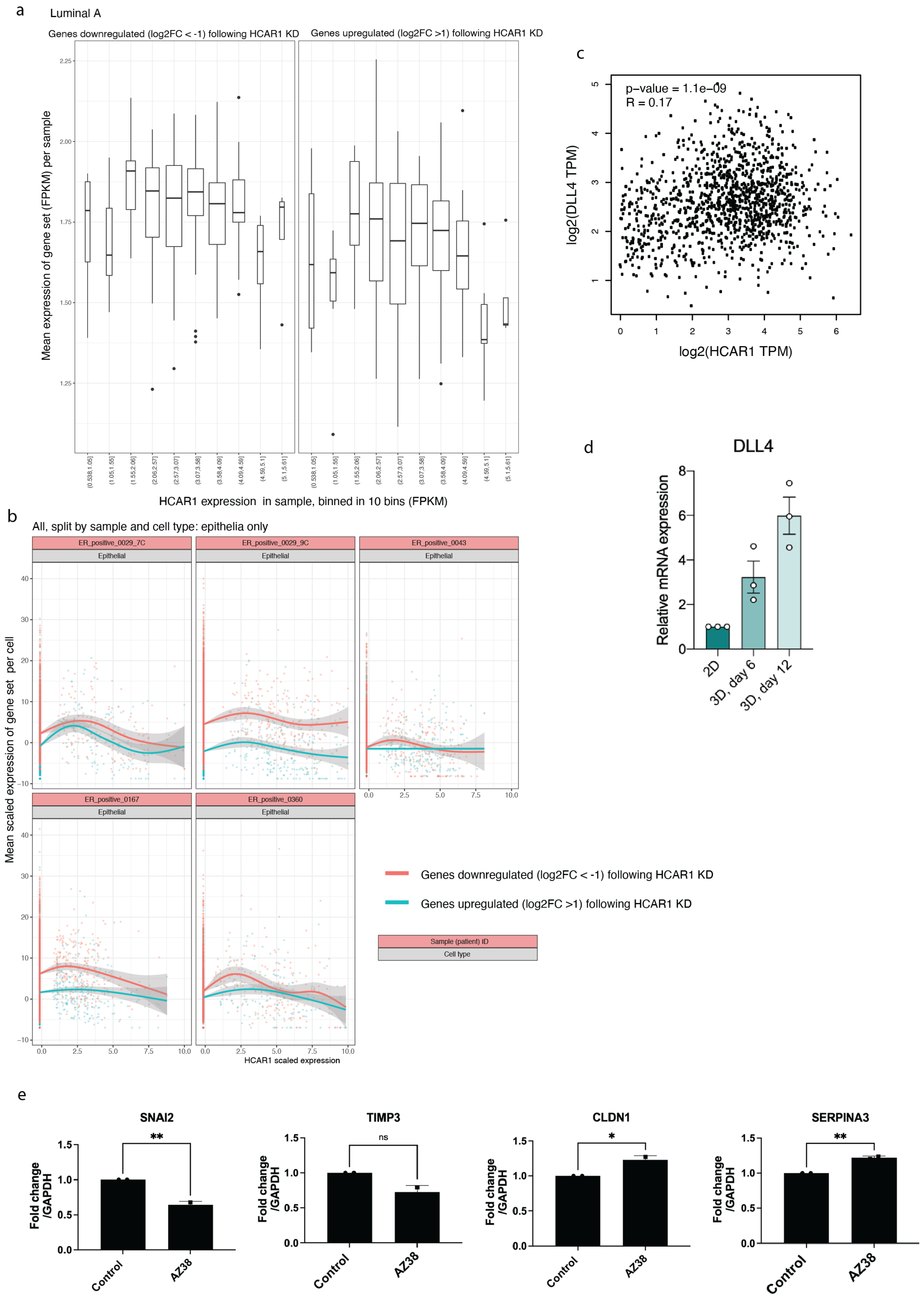
a
Luminal A
Genes downregulated (log2FC < -1) following HCAR1 KD
Genes upregulated (log2FC >1) following HCAR1 KD
Mean expression of gene set (FPKM) per sample
HCAR1 expression in sample, binned in 10 bins (FPKM)
b
All, split by sample and cell type: epithelia only
ER_positive_0029_7C
ER_positive_0029_9C
ER_positive_0043
ER_positive_0167
ER_positive_0360
Epithelial
Mean scaled expression of gene set per cell
HCAR1 scaled expression
Genes downregulated (log2FC < -1) following HCAR1 KD
Genes upregulated (log2FC >1) following HCAR1 KD
Sample (patient) ID
Cell type
c
p−value = 1.1e−09
R = 0.17
log2(DLL4 TPM)
log2(HCAR1 TPM)
d
DLL4
Relative mRNA expression
2D
3D, day 6
3D, day 12
e
SNAI2
TIMP3
CLDN1
SERPINA3
Fold change /GAPDH
Control
AZ38
**
ns
*
**

Supplementary figure 5. In situ analysis of spatial expression pattern of GPR81 regulated genes in MCF-7 spheroids

a Analysis of the expression of up- or down-regulated genes following HCAR1 depletion in NN cells and HCAR expression in bulk-seq TCGA RNA-seq data. Data is based on bulk RNA-seq from TCGA breast cancer samples (see Supplementary Methods). The X axis shows HCAR1 expression, binned in 10 bins. The Y axis show average expression of a given gene set from the same data. Gene sets are defined by genes that were upregulated or downregulated by HCAR1 depletion from our data (Table S3 and S4), but using a |log2FC| threshold of 1. Boxplots show the distribution of these means for each bin. **b** Analysis of the expression of up- or down-regulated genes following HCAR1 depletion in NN cells and HCAR expression in single-seq RNA-seq data. Data is based on single RNA-seq data from [4] and [5] (see Supplementary Methods). Only analysis from cells labelled 'Epithelia' from samples where HCAR1 showed variable expression are shown. Gene sets are defined by genes that were upregulated or downregulated by HCAR1 depletion from our data (Table S3 and S4), but using a |log2FC| threshold of 1. Each panel represents one sample (indicated in red). Dots represent values from individual cells. The X axis shows scaled HCAR1 expression values. The Y axis show the average scaled expression in the same cell across the respective gene set, indicated by color. A fitted trendline is added, using loess fitting, for each gene set. Grey color indicates the 95% confidence interval of the fit. **c** DLL4 mRNA expression in breast cancer patient tumors positively correlates with HCAR1 mRNA expression. Correlation performed in Gepia2 using all TCGA data for BRCA. P-value 1.1×10^{-9}. **d** DLL4 mRNA expression positively correlates with the increasing GPR81 expression in MDA-MB-231 cells, from 2D culture and with increasing spheroid growth in 3D.

Suppl. Fig. 6

In situ analysis of expression in MCF-7 spheroids

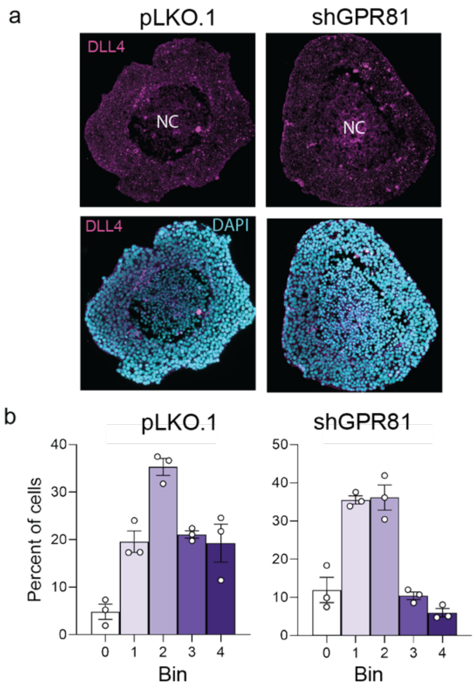

Co-clustering analysis of expression in MCF-7 GPR81sh spheroids

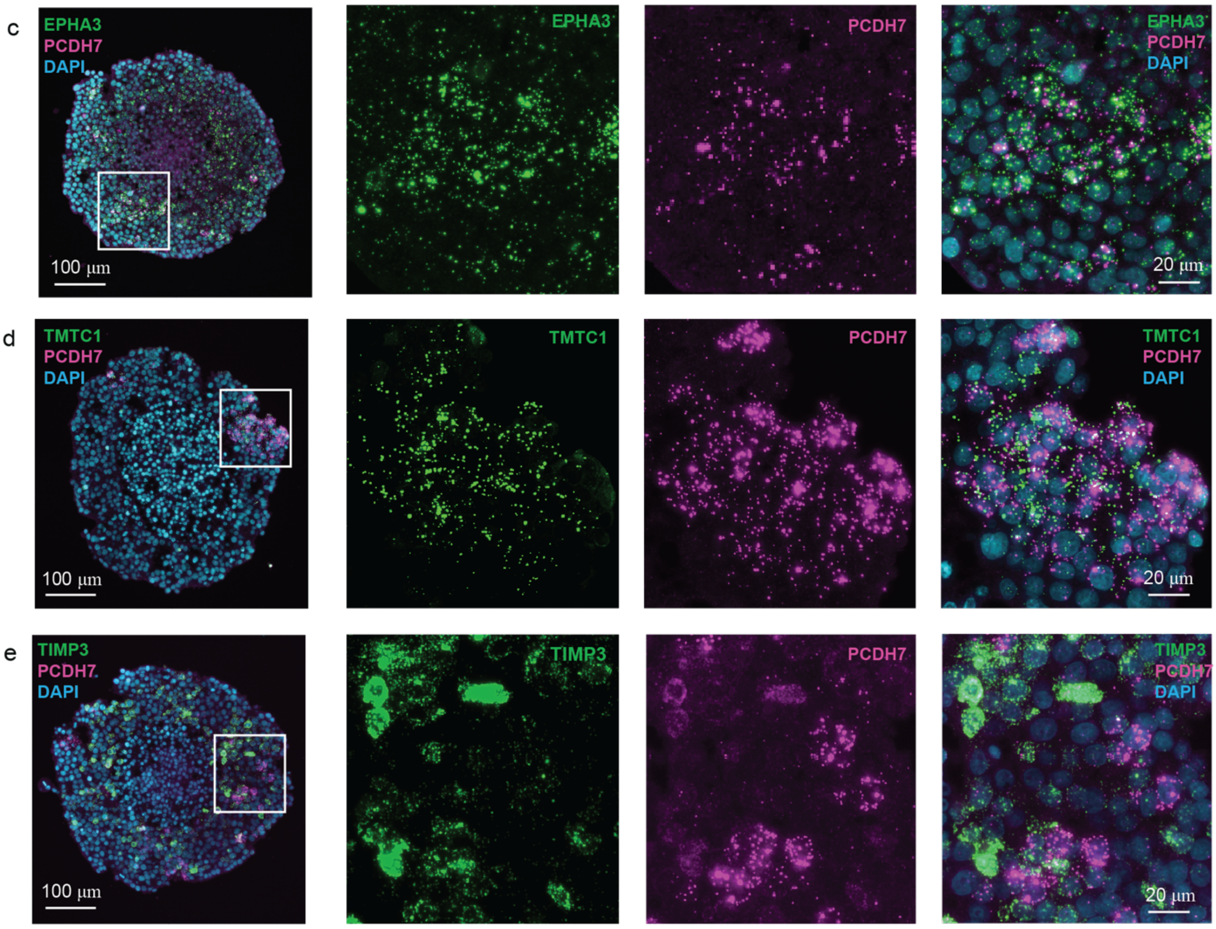

Supplementary figure 6. In situ analysis of spatial expression pattern of GPR81 regulated genes in MCF-7 spheroids

a-b Representative images (**a**) and corresponding quantification (**b**) of *in situ* hybridization of DLL4 in MCF-7 pLKO.1 and shGPR81 spheroids. The Y-axis shows % cells with a given bin distribution (for a detailed description, see Methods). **c-e**. Representative images of PCDH7 (magenta) localization in MCF-7 shGPR81 spheroids, co-stained for EPHA3 (**c**), TMTC1 (**d**) and TIMP3 (**e**) (green), detected by RNAscope. Representative of 3 biological n per condition. The first image in each row shows a whole spheroid, followed by higher magnifications of the boxed regions.

Suppl. Fig. 7

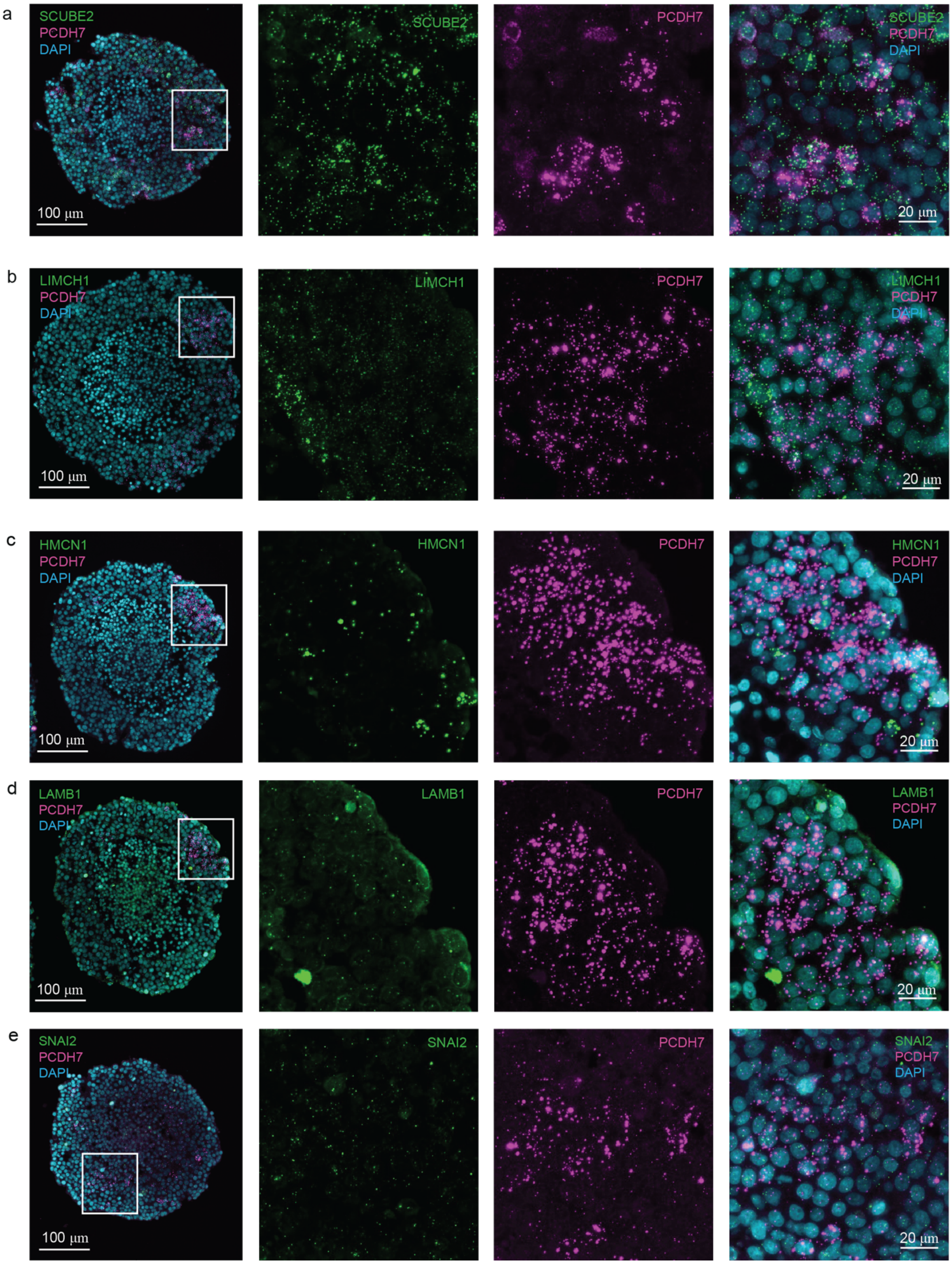
a
SCUBE2
PCDH7
DAPI
100 μm
SCUBE2
PCDH7
SCUBE2
PCDH7
DAPI
20 μm
b
LIMCH1
PCDH7
DAPI
100 μm
LIMCH1
PCDH7
LIMCH1
PCDH7
DAPI
20 μm
c
HMCN1
PCDH7
DAPI
100 μm
HMCN1
PCDH7
HMCN1
PCDH7
DAPI
20 μm
d
LAMB1
PCDH7
DAPI
100 μm
LAMB1
PCDH7
LAMB1
PCDH7
DAPI
20 μm
e
SNAI2
PCDH7
DAPI
100 μm
SNAI2
PCDH7
SNAI2
PCDH7
DAPI
20 μm

Supplementary Figure 7. In situ analysis of co-expression of PCDH7 in GPR81 KD MCF-7 spheroids

a-f. Representative images of PCDH7 (magenta) co-expression with SCUBE2 (**a**), LIMCH1 (**b**), HMCN1 (**c**), LAMB1 (**d**), and SNAI2 (**e**) (green) in GPR81 KD (shGPR81) spheroids, as detected by RNAscope. The first image in each row shows a whole spheroid, followed by higher magnifications of the boxed regions.

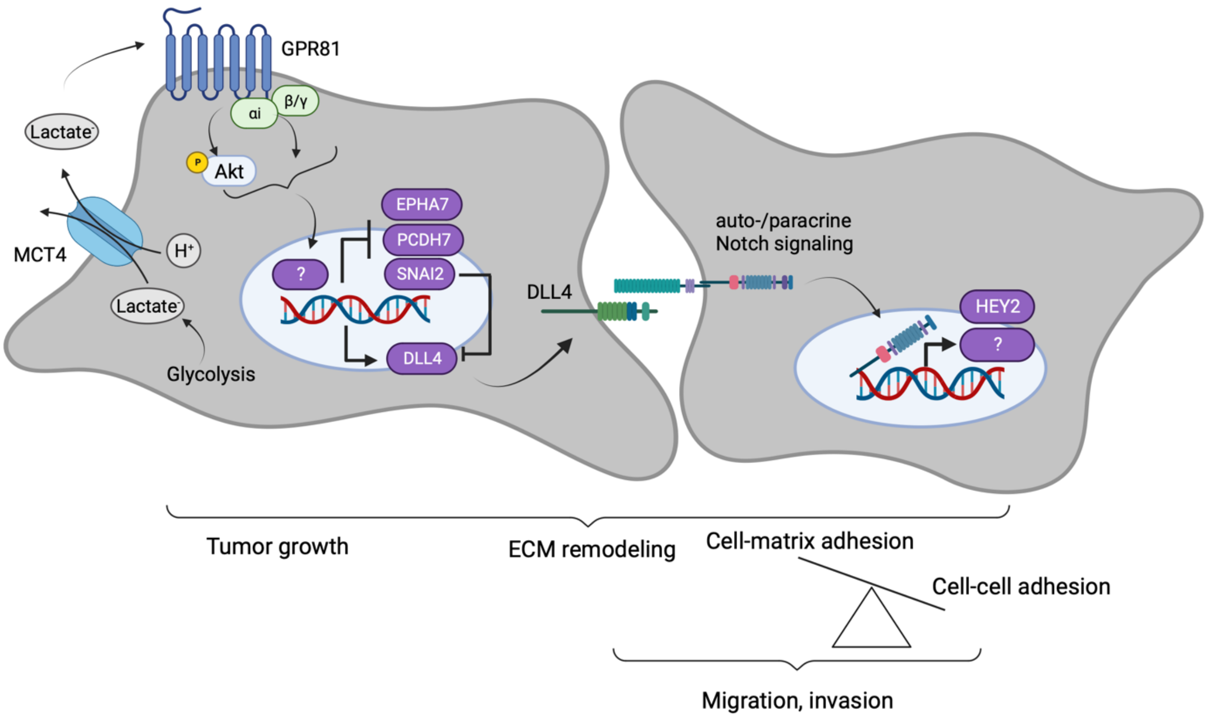

Supplementary Figure 8. Summary and working hypothesis

Lactate accumulation in the tumor microenvironment activates GPR81 signaling, leading to activation of downstream signaling events, including activating phosphorylation of Akt. One or more of these events elicit, via activation of yet unidentified transcription factor(s), to up- and downregulation of multiple genes. The genes downregulated by GPR81 signaling include EPHA7, PCDH7, and SNAI2, and those stimulated by GPR81 signaling include DLL4 (the latter likely in part due to the downregulation of its inhibitor SNAI2). The upregulation of DLL4 drives increased auto/paracrine Notch signaling, leading to regulation of Notch signaling-driven genes such as HEY2. Collectively, these events lead to increased tumor growth, ECM remodeling, cell-matrix/cell-cell adhesion balance, and increased cell migration and invasion. See text for details.

SUPPLEMENTARY TABLES

Supplementary Tables 1-7 are uploaded as excel sheets, and their titles and legends are provided in the main manuscript file.

Supplementary tables 8-11 are provided in full below.

Supplementary table 8. Sequences of primers employed.

Target	Forward (5'-3')	Reverse (5'-3')
ALDH3A1	GGAGAGGCTGTGTCAAAGG	GGCGTTCCATTCATTCTTGTG
ATCB	AGAGCTACGAGCTGCCTGAC	AGCACTGTGTTGGCGTACAG
ATP10D	TGAACAATTTCACAGAGCTGCC	GCTTCTACCAAAGGTACCCAGT
BCAT1	CTACGACCCTTGGGATCTGC	GCATCCGTTACTGCAAACCCCAGT
CA2	CAGGACAAAGCAGTGCTCAAG	GTCCATCAAGTGAACCCCAGT
CCDC80	GAAGACGTACCAGCCCATTT	GGGAAGGATACCAGGATTTGAC
CDH3	TGACCACAAGCCCAAGTTTAC	TAAGCAACCACCCCATTGTAG
CD36	TGTGCAAAATCCACAGGAAG	GCCACAGCCAGATTGAGAAC
DLL4	GGCCAACTATGCTTGTGAATGTC	ACCTCGGTTCAGGCACTGTC
DPYD	GGTGGTGATGTCGTTGGTTT	GCAGAAACGGAAGCTCCATA
EPHA3	TGGACTGCCCAGCTGCC	CCTTGCGGCTGCACTGGTGA
EPHB6	CCGCTACTATGACCAGGCAG	GCTCCCCTTGAGGAAGTGTC
GPR81	CTGGCATGACATCATGTTCC	CTTCTTCATCCGAGCCTGTC
GPR109A	GCGTTGGGACTGGAAGTTTG	GCGGTTCATAGCCAACATGA
GPR109B	CGTGATGGACTACTATGTGCG	ATTTGCAGGGCCATTCTGGAT
HMCN1	CTGAGGAAATTCCCGAGGGG	GTCACTGGGCCAATTTCTGG
IFIT2	GGACCCTGAAAACCCTGAAT	TGTGGCTAATTTAAAGCCATCC
LIMCH1	CTTCTCCGAGGCGCAGAA	CTGTCCGAAAATCTTTATCACCAA
PADi2	CTGCTGGAGAAGGCGCA	GGGGCCGCGCTGTAG
PCDH7	GACTCTGGGCGTCTCTGAAG	CTCACCTCCGACTCTGCTCA
PCDHA10	TCAGTCTATTGGAGGGGACC	TCCTGCCTCTGGTTCTGGT
PPARG	CCAGAAGCCTGCATTTCTGC	GTGTCAACCATGGTCATTTCGTT
PRG4	GTTTCATCTCAAGAGCTTTCCTGT	TGTGGGATTATGCACTTCTGC
SCUBE2	TTCACCCACTGTGGAGACAC	CAGTGGAGCTTGTACCCAGG
SERPINA3	TAGCAGTCTCCCAGGTGGTC	GGCCTGTTGAAACGCACAAT
SLC6A14	AAATCGTCTGGCAAGGTGGT	CCAGAGTGGCACCTCGTACT
SNAi2	CGAACTGGACACACATACAGTG	CTGAGGATCTCTGGTTGTGGT
SPARC1	TCGGCATCAAGCAGAAGGAT	ATTGGGGGAAACACGAAGGG
SPANXA2	AGGCCAACGAGATGATGCC	GATTCTGTTCTCTCGGGCGT
STING1	CTGGGATAAACTGCCCCAGCA	GTCATCTGCAGGTTCCGCT
TBP	GAGCTGTGATGTGAAGTTTCC	TCTGGGTTTGATCATTCTGTAG
TGFB2	GCGACGAAGAGTACTACGCC	TGGCATCAAGGTACCCACAG
TIMP3	GTGCAACTTCGTGGAGAGGT	CAGGTAGTAGCAGGACTTGATCTTG
TMTC1	GCTGTTTCTATTGGCCTTTCTC	TGTCTCTTTCACCAGCATCG
TXNIP	ACTCGTGTCAAAGCCGTTAGG	TCCCTGCATCCAAAGCACTT
WFIKKN2	TCGGTGTGTCGATGGAAGAG	TCATAGGTCTCACACTCCAGA

Supplementary table 9. List of siRNAs employed.

Name	Company	Cat. no
esiRNA targeting FLUC (esiRNA1)	Sigma Aldrich	EHUFLUC-50UG
MISSION® siRNA Universal Negative Ctrl. #1	Sigma Aldrich	SIC001
DLL4	Sigma Aldrich	SASI_Hs02_00352665
EPHA7	Sigma Aldrich	SASI_Hs01_00154992
GPR81	Sigma Aldrich	SASI_Hs01_00101136
PCDH7	Sigma Aldrich	SASI_Hs01_00120976
SPARC	Sigma Aldrich	SASI_Hs01_00065300
**shRNAs**		
**Name**	**Company**	**Cat. no**
MISSION® GPR81 shRNA bacterial glycerol stock	Sigma Aldrich	TRCN000008941<br>#1: NM_032554.2-1217s1c1<br>#2: NM_032554.2-2200s1c1
MISSION® pLKO.1-puro Empty Vector Control Plasmid DNA	Sigma Aldrich	SHC001

Supplementary table 10. List of antibodies employed.

**Primary antibodies**	**Company**	**Reference**
α-tubulin	Sigma-Aldrich	#T5168
AKT	Cell Signaling	#CS-9272
pAKT	Cell Signaling	#CS-4060
β-actin	Sigma	#A5441
DCTN1	BD Biosciences	#BD-610474
DLL4	Cell Signaling	#CS-96406
Epha7 (D1C3K)	Cell Signaling	#64801
ERK 1/2	Cell Signaling	#CS-9102
Phospho-ERK	Cell Signaling	#CS-9101
GPR81	Abcam	Ab124010
GPR81	Novus Biologicals	NLS2095
Ki67	Cell Signaling	#9449
PCDH7	Abcam	ab139274
Phospho-Rb (Ser807/811)	Cell Signaling	#CS-9308
SPARC	Thermo Scientific	#33-5500
**Secondary antibodies**	**Company**	**Reference**
Goat-anti mouse Alexa Fluor® 488	Invitrogen	A32723
Goat-anti rabbit Alexa Fluor® 568	Invitrogen	A-11011
Goat-anti rabbit Alexa Fluor ® 488	Invitrogen	A32731

Supplementary Table 11. List of in situ probes and fluorophores employed.

Name	Company	Cat. no
Hs-HCAR1-O3	Advanced Cell Diagnostics	855851
Hs-EPHA7	Advanced Cell Diagnostics	464661
Hs-EPHA7-C2	Advanced Cell Diagnostics	464661-C2
Hs-PCDH7-C2	Advanced Cell Diagnostics	527131-C2
Hs-DLL4-C3	Advanced Cell Diagnostics	603001-C3
Hs-SPARC-C3	Advanced Cell Diagnostics	425111-C3
Hs-Epha3-C3	Advanced Cell Diagnostics	1072211_C3
Hs-Tmtc1	Advanced Cell Diagnostics	862891
Hs-Timp3_C3	Advanced Cell Diagnostics	433101-C3
Hs-Snai2	Advanced Cell Diagnostics	554581
Hs-Scube2	Advanced Cell Diagnostics	479751
Hs-Hmcn1	Advanced Cell Diagnostics	433151
Hs-Lamb1	Advanced Cell Diagnostics	550451
Hs-Limch1-C1	Advanced Cell Diagnostics	1153991-C1
Positive Control Probe_Hs	Advanced Cell Diagnostics	313901
Negative Control Probe_Hs	Advanced Cell Diagnostics	320871
Opal520 Reagent	Akoya Biosciense	FP1487001KT
Opal690 Reagent	Akoya Bioscience	FP1497A
Opal570 Reagent	Akoya Bioscience	FP1488001KT
